# Network Pharmacology Approach to Investigate the Mechanism of Modified Liu Jun Zi Decoction in the Treatment of Chronic Atrophic Gastritis

**DOI:** 10.1155/2022/7536042

**Published:** 2022-06-17

**Authors:** Ming Zhou

**Affiliations:** Nanjing University of Chinese Medicine, School of Chinese Medicine, School of Integrated Chinese and Western Medicine, Nanjing 210023, China

## Abstract

Although modified Liu Jun Zi decoction (MLD) has favorable outcomes for chronic atrophic gastritis (CAG) in clinics, the identification of its active ingredients and the molecular mechanism of pharmacology are still unknown and need to be solved urgently. In the study, we screened 170 active components of MLD based on oral bioavailability ≥30% and drug-likeness ≥0.18 via the TCMSP platform. We further establish a dataset containing 315 CAG targets from PharmGkb, GeneCard, OMIM, DrugBank database, and Therapeutic Target database. Network pharmacology found that there are 110 active components of MLD and 26 potential targets for CAG in the “ingredient-target” network. The results of gene ontology analysis show that these targets are involved mainly in reactive oxygen species metabolic process, regulation of vasculature development, and T cell activation. KEGG pathways analysis indicates that these signaling pathways in the treatment of CAG include HIF-1 signaling pathway, neurodegeneration-multiple diseases pathway, MAPK signaling pathway, and PI3K-Akt signaling pathway. Finally, docking of the active component quercetin and clinical medicine Omeprazole with the core targets was carried out. We found that quercetin, a crucial active ingredient in MLD, has good binding activity with potential targets of CAG, and its molecular conformation is stable, which is better than the binding energy of Omeprazole. So, the active ingredients of MLD exhibit good potential drugs for the treatment of CAG.

## 1. Introduction

Chronic atrophic gastritis (CAG) is frequently an inflammatory disease of the digestive system [1], with epigastric pain, fullness, belching, and anorexia as the main clinical manifestations. The pathological changes include the decrease of gastric mucosal glands, intestinal metaplasia, or atypical hyperplasia. Moreover, it is often regarded as a high-risk factor for gastric cancer [2–4]. CAG is on the rise in the normal population in China, in which the incidence accounts for about 20% in the tested population. CAG with intestinal metaplasia has a canceration rate of 13% and the average time of canceration is 3.5 years. The canceration rate of atypical hyperplasia is 18.1% and the average time of canceration is 1.5 years. “Inflammation-mediated carcinogenesis” is a hot spot of present research on the mechanism of tumorigenesis. Delaying or reversing the “inflammation-mediated carcinogenesis “ of CAG will be an important idea to prevent the occurrence and development of gastric cancer.

Clinically, CAG could be divided into two categories: autoimmune gastritis (AG) and multifocal atrophic gastritis (MAG) [5, 6]. MAG is the most common in China and AG is rare. *Helicobacter pylori* infection is the most common cause of CAG, and more than 80% of CAG patients have detected positive for *H. pylori* sera. In addition, environmental factors, bile reflux, smoking and alcohol consumption, diet, and drugs are also a part of the risk factors for CAG. In the clinical practice, modern medical diagnosis of CAG is mainly based on the results of gastroscopy and biopsy. The red and white gastric mucosa can be seen in the stomach of CAG patients under endoscopy, and the white mucosa is the main one. Compared with normal gastric mucosa, the gastric mucosa vessels of CAG patients were exposed, the folds were flattened or disappeared, and there were small hyperplastic particles or larger nodules. Moreover, scattered patches of erosion and blood spots are seen in the patient's gastric mucosa.

So far, the etiology and specific pathogenesis of CAG are still not very clear, and there is a lack of effective drugs in clinical treatment. Clinically, a compound drug therapy is still dominant and has been extensively used for treating CAG. Moreover, the principle of individualization should be followed [7–10]. Up to now, Western medicine still lacks effective means and drugs for the treatment of CAG, while Chinese medicine has unique advantages.

Chronic atrophic gastritis has no definite disease name in ancient Chinese medicine literature. But, according to the fullness, epigastric pain, and other symptoms, it can be classified as “distention and fullness,” “stomach upset”, and “epigastric pain” in traditional Chinese medicine. In Huangdi Neijing, Shanghan Lun, Jingyue Quanshu, and other ancient medical books, the pathogenesis and syndrome differentiation of this disease are also clearly indicated. Modern scholars have systematically sorted and analyzed the literature on the diagnosis and treatment of CAG by famous veteran teran doctors of TCM and found that the most frequent understanding of its etiology and pathogenesis is spleen qi deficiency. On the whole, they believe that the pathogenesis of this disease is mainly based on deficiency in origin and excess in superficiality. For example, qi stagnation or phlegm dampness can be seen on the basis of spleen and stomach weakness. Syndrome differentiation and treatment is also quite effective. It has obvious effect in reducing symptoms, delaying, or reversing pathological changes and can provide important schemes and strategies for the prevention of gastric cancer.

Six Chinese herbs were composed in Liu Jun Zi decoction including *Panax Ginseng C. A. Mey* (Renshen), *Atractylodes macrocephala Koidz* (Baizhu), *Poria cocos* (Schw.) *Wolf* (Fuling), *Licorice* (Gancao), *Citrus Reticulata* (Chenpi), and *Arum ternatum Thunb* (Banxia). The whole prescription has the effects of invigorating the spleen and stomach, reducing phlegm and regulating qi, and it was also employed to treat various digestive diseases such as gastritis, duodenitis, and inflammatory bowel diseases. In addition, this formula is also extensively used to treat gastric cancer and the side effects of radiotherapy and chemotherapy, which can improve the prognosis of patients. It is already in clinical trials in the United States and was approved for the European Union market by the German Food and Drug Administration in 2019. At present, Japan is at the forefront of the world in the research and marketization of this formula.

Classical western medicine is a compound corresponding to a target to treat a disease. Its advantage is that the drug composition and pharmacological mechanism are clear. Traditional Chinese medicine, especially prescription drugs, often contains multiple active ingredients, which target multiple targets and work in multiple pathways. Traditional Chinese medicine prescription can achieve a comprehensive therapeutic effect, resulting in its complex pharmacology and difficulty to distinguish. With the continuous development of bioinformatics and system biology, the thinking mode of network pharmacology is more integrated and its predictive ability is more powerful in the research of traditional Chinese medicine.

Hiroyuki et al. [11] found 32 active chemical components in Liu Jun Zi decoction with jujube and ginger. Xuchun Su used high-performance liquid chromatography to analyze the four main active ingredients in Liu Jun Zi decoction, including *Codonopsis pargynoside*, *Atractylodes lactone II,* hesperidin, and *Pinellia ternata* total alkaloids, and found that the content of active ingredients in Liu Jun Zi decoction increased significantly after processing, which could enhance the therapeutic effect. Gu et al. used liquid-mass spectrometry (LC-MS) to analyze the three active components in Liu Jun Zi decoction, including *Atractylodes lactone III,* quercetin, and fuling acid in Liu Jun Zi decoction, and found that only *Atractylodes lactone *III was detected in serum. Based on the integrated thinking of network pharmacology and strong predictive ability, Yang et al. analyzed 491 compounds contained in Sijunzi decoction (*Codonopsis pilosula* (Franch.) Nannf., *Poria cocos* (Schw.) Wolf, *Atractylodes macrocephala* Koidz, and licorice) and screened 123 active components corresponding to 78 targets of CAG. Further GO and KEGG analysis showed that Sijunzi decoction mainly regulates CAG by regulating cell inflammation, proliferation, apoptosis, and metabolism, which provides a basis for clarifying the mechanism of action of Sijunzi decoction in the treatment of CAG and conducting experimental verification. In view of the wide application of Liu Jun Zi decoction in the treatment of CAG, the mechanism of its active components needs to be clarified urgently. However, up to now, no systematic network pharmacological analysis of Liu Jun Zi decoction has been established for CAG.

In the study, the modified Liu Jun Zi decoction is based on Liu Jun Zi decoction with the addition of two traditional Chinese medicines, which are *Coicis semen* (Yiyiren) and *Fritillariae thunbergii Bulbus* (Zhebeimu). *Coicis Semen* is a kind of the medicine-food herb, known as the “King of Gramineae.” Esters, unsaturated fatty acids, sugars, triterpenoids, and lactams are the main active components of *Coicis semen*. In addition to the good effect of invigorating spleen and expeling dampness according to traditional Chinese medicine theory, the coix seed also has the functions of enhancing body immunity, regulating intestinal flora, regulating glucose and lipid metabolism, analgesia and anti-inflammatory, anticancer, and antioxidation activities. Moreover, clinical adverse reactions are less. *Fritillaria thunbergia Bulbus* is a traditional Chinese medicine for resolving phlegm and relieving cough, and it is said in the Mingyi Bielu that it can “treat the fullness under the heart.” According to the clinical experience of Mr. Xu Jingfan, a famous traditional Chinese medicine, *Fritillaria thunbergii* is often effective for some patients with difficult stomach diseases. Moreover, modern studies have found that there are alkaloids, polysaccharides, and total saponins in *Fritillariae Thunbergii*, among which alkaloids are the core active components. It has antitussive, expectorant, analgesia, antiulcer, antibacterial, antitumor and anti-inflammatory activities. Therefore, the addition of *Coicis semen* and *Fritillaria Thunbergia Bulbus* on the basis of Liu Jun Zi decoction is a reasonable compatibility based on the comprehensive consideration of the etiology and pathogenesis, clinical symptoms, pathological manifestations, and drug safety of CAG and has an ideal clinical effect. In this project, we analyzed the active components of modified Liu Jun Zi decoction. A series of analysis were conducted to explore the mechanism of modified Liu Jun Zi decoction in the treatment of CAG by multicomponent, multitarget and multichannel means, such as target analysis, network pharmacological analysis, pathway analysis of targeted regulation, analysis of CAG differential gene expression, molecular docking analysis of active components, compound cell assay, clinical treatment guides, and result analysis.

## 2. Methods

### 2.1. Chinese Herbal Ingredient Collection and Screening

**Figure 1:**
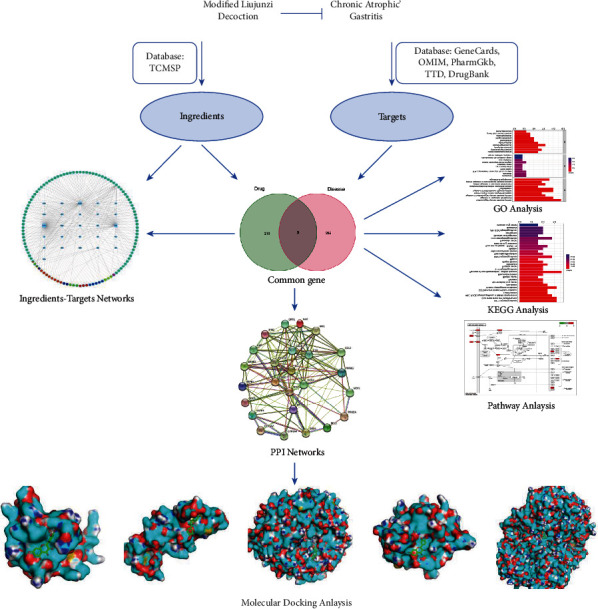
Schematic illustration showing the network pharmacology to investigate modified Liu Jun Zi decoction ingredients for chronic atrophic gastritis treatment.

As shown in Figure 1, we used the network pharmacology to investigate modified Liu Jun Zi decoction ingredients for chronic atrophic gastritis treatment. TCMSP, a database of system pharmacology for drug discovery from herbal medicines, records Chinese herbal ingredients, effect targets, and relative diseases. From this database, we collected the chemical composition information of Chinese herbal medicine in the modified Liu Jun Zi decoction (MLD) including Renshen (*Panax ginseng C. A. Mey*), Baizhu (*Atractylodes macrocephala Koidz*), Fuling (*Poria Cocos(Schw.) Wolf*), Gancao (*Licorice*), Chenpi (*Citrus reticulata*), Banxia (*Arum ternatum Thunb*), Zhebeimu (*Fritillariae thunbrgii Bulbus*), and Yiyiren (*Coicis semen*). The candidate ingredients of MLD were screened with oral bioavailability (OB) ≥ 30% and druglikelihood (DL) ≥ 0.18 [12, 13]. Furthermore, the potential targets of MLD were retrieved from TCMSP database. For the next assay, we generated the potential targets information of MLD with a gene symbol (Supplementary Table 1) with the UniProt database (https://www.uniprot.org/uniprot).

### 2.2. Chronic Atrophic Gastritis-Related Target Genes and Affiliated MLD Active Ingredients

Chronic atrophic gastritis-related target genes were searched and screened using atrophic gastritis as a keyword from five databases: OMIM database (https://www.omim.org), Therapeutic Target database (http://db.idrblab.net/ttd/), PharmGkb database (https://www.pharmgkb.org), DrugBank database (https://www.drugbank.ca), and GeneCard database (https://www.genecards.org). Then, the search target genes combined and deleted the duplicate genes to form a cluster of potential target information of CAG). Furthermore, the active ingredient targets of MLD and the disease targets of CAG were analyzed by drawing Venn diagrams with “venn” *R* package. The intersection target genes of MLD and CAG were collected as the common genes, which might be the potential targets of MLD for CAG treatment).

### 2.3. Network Analysis of MLD Active Ingredients and CAG Target Genes

The network of active ingredient of MLD and the disease targets of CAG were constructed by Cytoscape [14]. CytoNCA was executed to analyze the topology properties of the network, consisting of betweenness centrality (BC), closeness centrality (CC), degree centrality (DC), eigenvector centrality (EC), local average connectivity-based method centrality (LAC), and network centrality (NC). In this section, the degree refers to the number of direct neighbors of a node. The nodes in the network represented the active component or target, and the edges represented their interaction. The degree of nodes refers to the number of direct neighbors from a node. It means that the greater the number is, the greater the influence is in the network.

### 2.4. Protein-Protein Interaction (PPI) Network Construction and Core Gene Screening

In this study, a lot of protein interactions were collected by STRING database (https://string-db.org/cgi/input.pl). Common target genes of MLD and CAG were imported into the STRING database for constructing PPI network with a confidence level of 0.7 and to hide disconnected nodes [15, 16]. The TSV format of the updated results was imported into Cytoscape 3.8 and CytoNCA Apps software was employed to analyze the topology properties of the interaction network by calculating degree centrality (DC), betweenness centrality (BC), closeness centrality (CC), eigenvector centrality (EC), network centrality (NC), and local average connectivity-based method centrality (LAC) [17]. As we all know, these six parameters indicate the property of the nodes in the interaction network. A node with high DC, BC, CC, EC, NC, and LAC values means that it plays a highly important role in the network. So, we collected these targets above the median as the core targets and screened them twice to obtain the core target genes [18]. Further, MCODE was used to screen the modules with significant interactions for constructing a network pharmacology map of Chinese herbal-effect ingredients-core CAG target genes.

### 2.5. Function Enrichment Analysis by GO and KEGG

The Entrez gene ID of the core target genes were extracted through org.Hs.eg.db, and then the GO function enrichment was carried out by ClusterProfiler, org.Hs.eg.db, enrichplot, and ggplot2 in *R* software. Subsequently, the KEGG pathway was enriched by pathview package. The screening criteria were qvalue-cutoff = 0.05. The top 30 items with the highest enrichment were selected and displayed in bar graphs in order to provide reference information for subsequent experiments.

### 2.6. Preparing the Structure Files of Drug-Like Ingredients and CAG Target Protein

The molecular structure file of Omeprazole and core drug-like ingredients from network pharmacology were extracted from PubChem database (https://pubchem.ncbi.nlm.nih.gov). The sdf file of 2D compounds were converted to the mol2 files of 3D compounds with minimum free energy through Chem3D software. Subsequently, the crystal structure files of CAG target proteins were downloaded from RCSB PDB database (https://www.rcsb.org). The PyMOL-2.4.0 software was used to remove water molecules and small molecule ligands from the crystal structure of proteins.

### 2.7. Molecular Docking

In order to make a molecular docking compound with proteins, we first add hydrogen for the receptor protein to prepared the PDBQT file and determined the active pocket of protein by Autodock1.1.2. Subsequently, the receptor proteins and ligands were submitted to Chimera v1.6.2 for structural preparation and get conformations with energy minimization. We set the parameters based on the above active pocket for molecular docking to achieve minimum binding free energy in vina software. Finally, we can visualize the results of molecular docking by PyMOL2.4.0. According to the minimum binding free energy of compound and receptor protein, we can estimate the capacity of inhibitory activity.

## 3. Results

### 3.1. Screening of Candidate Active Ingredients and Targets in MLD

We achieved 170 candidate active ingredients of MLD from the TCMSP database with the screening conditions with oral bioavailability (OB) ≥ 30% and druglikelihood (DL) ≥ 0.18. Among them, 22 candidate ingredients were from *Panax ginseng C. A. Mey* (Renshen), 7 ingredients from *Atractylodes macrocephala Koidz* (Baizhu), 7 ingredients from *Fritillariae thunbergii Bulbus* (Zhebeimu), and 13 ingredients from *Arum ternatum Thunb* (Banxia). As for *Citrus reticulatae* (Chenpi), there were 5 candidate ingredients in the specific Chinese herb. *Poria cocos* (Schw.) Wolf (Fuling) had 15 candidate ingredients. In addition, Licorice (Gancao) had 92 candidate ingredients and *Coicis semen* (Yiyiren) had 9 candidate ingredients. Furthermore, a total of 6286 target genes were in the 8 Chinese herbs of MLD. There were 748 in *Panax ginseng C. A. Mey* (Renshen), 774 in *Atractylodes macrocephala Koidz* (Baizhu), 110 in *Fritillariae thunbergii Bulbus* (Zhebeimu), 1302 in *Arum ternatum Thunb* (Banxia), 479 in *Citrus reticulatae* (Chenpi), 121 in *Poria cocos* (Schw.) Wolf (Fuling), 2506 in Licorice (Gancao) and 246 in *Coicis semen* (Yiyiren), respectively. Finally, we connected the candidate ingredients and MLD targets to obtain 2077 candidate ingredient targets of MLD (Supplementary Table S1).

### 3.2. Download Targets of CAG and Screening the Effective Active Ingredients

We searched the target genes in five disease databases, PharmGkb, TTD, GeneCard, OMIM, and DrugBank, by the key words of “chronic atrophic gastritis,” as well as ultimately got 315 target genes of CAG (Supplementary Table S2). We then convert the gene names to gene symbols for the 2077 targets of MLD using the UniProt database, null and repetitive targets were deleted. Finally, we connected the CAG targets and the ingredient targets of MLD to obtain 110 effective active ingredients of MLD and 26 CAG targets, as shown in Supplementary Table S3 and Supplementary Table S4.

### 3.3. Constructing the MLD Ingredient-CAG Target Network

**Figure 2:**
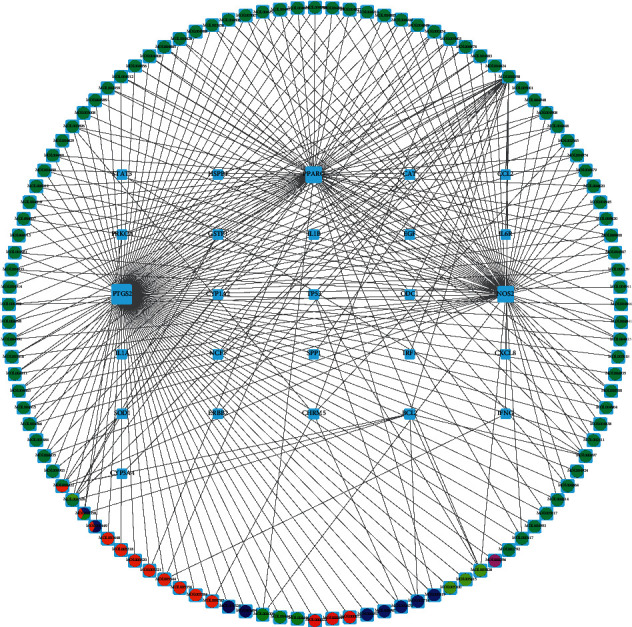
Ingredient-target network of modified Liu Jun Zi decoction for chronic atrophic gastritis. Molecules on the circle nodes are the main active ingredients of modified Liu Jun Zi decoction, including green from Gancao (Licorice), aqua from Zhebeimu (*Fritillariae Thunbergii Bulbus*), lime from Chenpi (*Citrus reticulatae*), blue from Banxia (*Arum ternatum Thunb*), navy from Yiyiren (*Coicis semen*), red from Baizhu (*Atractylodes macrocephala Koidz*), purple from Fuling (*Poria cocos(Schw.) Wolf*), and orange from Renshen (*Panax ginseng C A. Mey*). The cyan rectangle in the circle center is the potential target genes for treating chronic atrophic gastritis with modified Liu Jun Zi decoction.

We mapped the 110 active ingredients of MLD to 26 CAG targets to generate 286 terms of ingredient targets. These terms were imported into Cytoscape 3.8 to put up a visual MLD ingredient-CAG target network (Figure 2). The MLD ingredient-CAG target network showed that 3 CAG-related targets were with a degree value higher than 10, including PPARG (66), NOS2 (67), and PTGS2 (108). The degree means the number of direct neighbors for the node. It is known as the greater the degree is, the greater the influence is. PTGS2 is associated with 108 of the 110 active compounds in MLD and is the first core target. Among the 110 active compounds, 3 active drug components were with a degree value higher than 5, including MOL000098 (quercetin, 22), MOL000422 (kaempferol, 7), and MOL004328 (naringenin, 6). MOL000098 was associated with 22 of the 26 CAG core target genes. Different nodes represent the active constituents of MLD or the target of CAG. These results indicated that MLD has multicomponent and multitarget characteristics to treat CAG.

### 3.4. PPI and Core Gene Analysis

**Figure 3:**
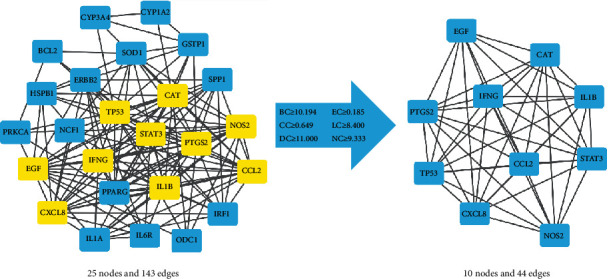
PPI network screening for core genes.

The 26 CAG core target genes were imported into the STRING database to gain the interaction relationship. The CHRM5 was not associated with other targets and was eliminated. Ultimately, the interaction network has 25 nodes and 143 edges (Figure 3(a)). We further analyze the network of PPI based on betweenness centrality (BC), closeness centrality (CC), degree centrality (DC), eigenvector centrality (EC), local average connectivity-based method centrality (LAC), and network centrality (NC) parameters by using CytoNCA plugin. The results of second screening, with the threshold of BC ≥ 10.194, CC ≥ 0.649, DC ≥ 11, EC ≥ 0.185, and LAC ≥ 8.4, were 10 nodes and 44 edges, including EGF, CAT, PTGS2, IFNG, IL1B, TP53, CCL2, STAT3, CXCL8, and NOS2 (Figure 3(b)).

### 3.5. Gene Ontology (GO) Enrichment Analysis

**Figure 4:**
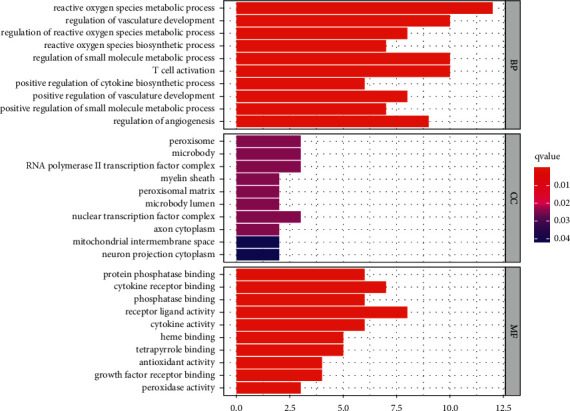
GO enrichment of common target genes for chronic atrophic gastritis treatment with modified Liu Jun Zi decoction active ingredients.

The biological processes (BP), cell components (CC), and molecular function (MF) of 26 core targets (*P* < 0.05) were analyzed, and the results showed that the BP were mainly involved in the metabolism and regulation of reactive oxygen species, the regulation of angiogenesis, and T cell activation, which were closely related to biological function. The CC were involve peroxisomes, microbodies, and nuclear transcription factor complexes. MF was mainly enriched in ligand receptor activation, phosphorylation, cytokine activation, etc. As shown in Figure 4, the top 30 GO terms were screened with the cutoff criteria of *P* < 0.05.

### 3.6. KEGG Pathway Enrichment Analysis

**Figure 5:**
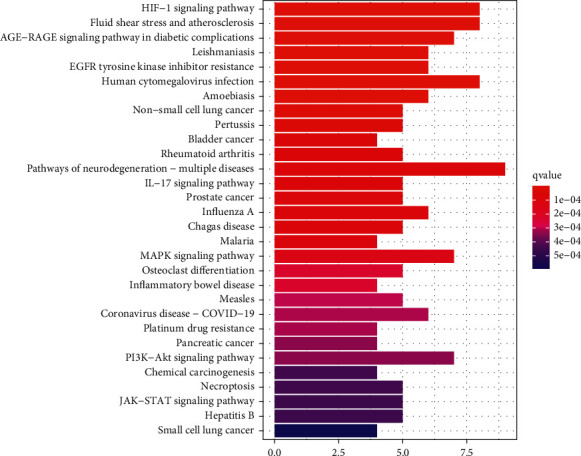
KEGG enrichment of common target genes for chronic atrophic gastritis treatment with modified Liu Jun Zi decoction active ingredients.

KEGG enrichment analysis was carried out for 26 targets of MLD. The results suggested that these targets were chiefly enriched in HIF-1 signaling pathways, pathways of neurodegeneration-multiple disease, MAPK signaling pathways and PI3K-Akt signaling pathways, etc. These classic signaling pathways play a vital function in the development of CAG. The top 30 KEGG analysis results are shown in Figure 5, which suggested MLD can act on CAG through multiple pathways.

### 3.7. Target Path Analysis

**Figure 6:**
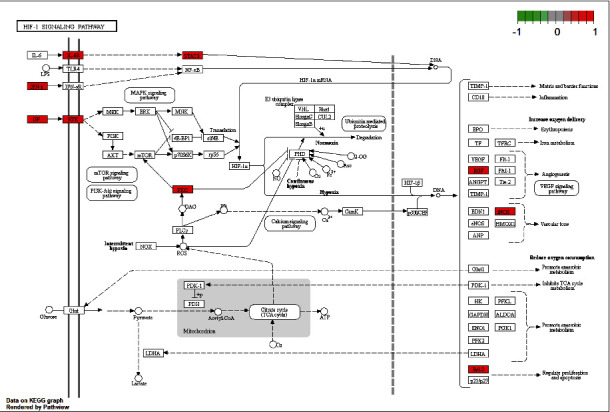
Pathway map of MLD in the treatment of chronic atrophic gastritis. Main target genes of MLD in the treatment of CAG are located in the HIF-1 signal pathway(hsa04066). Arrows represent the activation effect, T arrows represent the inhibition effect, and segments show the activation effect or inhibition effect.

**Figure 7:**
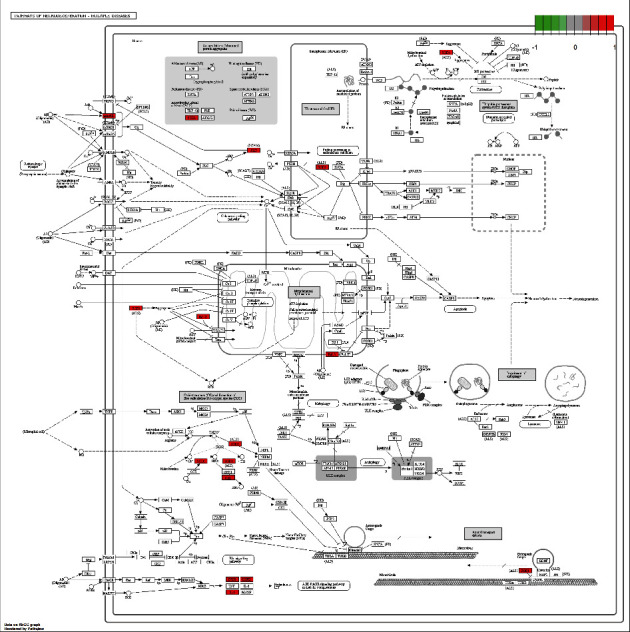
Pathway map of MLD in the treatment of chronic atrophic gastritis. Main target genes of MLD in the treatment of CAG are located in pathway of neurodegeneration-multiple diseases (hsa05022). Arrows represent the activation effect, T-arrows represent the inhibition effect, and segments show the activation effect or inhibition effect.

The *R* package “pathview” was used to map the treatment targets of MLD for CAG on selected pathway. The targets in the pathway were marked by white, and the targets of MLD for treating CAG were labeled in red. The results revealed that 8 effective targets of MLD were enriched in HIF-1 signaling pathways(hsa04066), including BCL2, PRKCA, NOS2, STAT3, EGF, IL6R, ERBB2, and IFNG (Figure 6). Moreover, the 9 effective targets of MLD, including PTGS2, CHRM5, BCL, PRKCA, SOD1, CAT, NOS2, IL1B, and IL1A, were enriched in pathways of the neurodegeneration-multiple disease (hsa05022, as shown in Figure 7). These findings suggested that these targets of MLD may be located in the pathways to execute its function for treating CAG.

### 3.8. Molecular Docking Analysis

**Figure 8:**
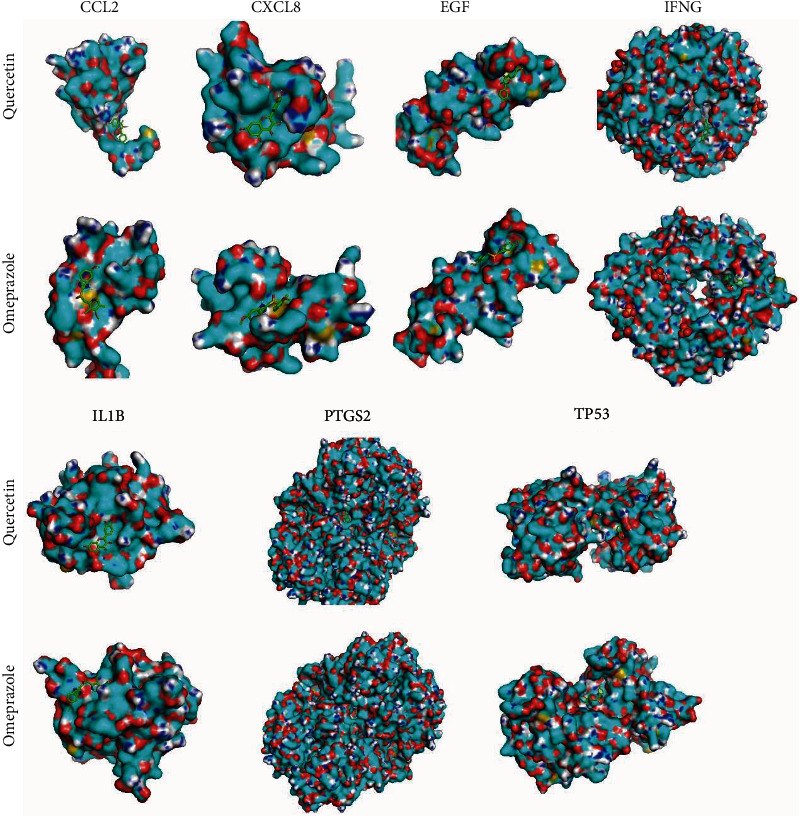
Molecular docking between MLD active compounds and CAG core targets.

We further analyzed the binding energy between MLD active compounds and CAG core targets by molecular docking (Figure 8). The co-crystallized structure of the 7 effective targets proteins were downloaded from the RCSB Protein Data Bank (http://www.pdb.org/) and modified using Autodock 4.2 software (La Jolla, CA, US) to remove ligands, add hydrogen, remove water, and optimize and patch amino acids. The protein preparation and binding pocket prediction were performed with Autodock. Subsequently, the receptor proteins and ligands were prepared with Chimera v1.6.2 prior to docking. The lower the binding energy of ligand and receptor, the more stable the conformation, and the greater the possibility of activity. The results reveal that quercetin possessed good binding activity with CCL2 (PDB ID: 1dok), CXCL8 (PDB ID : 5D14), EGFR (PDB ID : 1M17), PTGS2 (PDB ID: 5IKR), IL1B (PDB ID: 4i1b), TP53 (PDB ID: 3DCY), and IFNG (PDB ID: 1hig), which were potential targets of CAG, and the molecular conformational binding is stable. Its binding energy is better than that of Omeprazole, a western drug used in clinical treatment of CAG. The binding with potential targets is shown in Table 1.

## 4. Discussion

MLD has the effect of invigorating spleen and regulating gastrointestinal diseases. Clinically, it is widely used to treat chronic atrophic gastritis. In addition, accumulated evidence has shown that MLD can reduce the side effects after radiotherapy and chemotherapy, which can improve the prognosis and prolong the survival of patients. Nevertheless, the identification of active ingredients and pharmacological molecular mechanism of MLD is less known, which needs to be solved urgently. Furthermore, the systematic network pharmacological analysis of modified Liu Jun Zi decoction has not been established up to now.

In this study, the degree of quercetin, kaempferol, and naringenin was the highest in the ingredient-target network of MLD. Quercetin could inhibit inflammation and proteoglycan degradation through the downregulation of TNF-*α* and MMP-9 [19]. Kaempferol could also resist inflammatory response and decrease the inflammatory mediators via blocking the activation of p38, ERK, and NF- *κ*B signaling pathways [20, 21]. In addition, naringenin is a flavonoid compound that belongs to the subclass of flavanones. It is extensively distributed in several fruits and vegetables, as well as possesses antioxidant, antitumor, antiviral, antibacterial, anti-inflammatory, antiadipogenic, and cardioprotective effects [22]. These facts indicated that quercetin, kaempferol, and naringenin may be the most important components in the treatment of CAG by MLD.

The core targets of MLD for CAG treatment were CCL2, CXCL8, EGF, PTGS2, IL1B, TP53, and IFNG. The epidermal growth factor (EGF) is a potent promotor for carcinogenesis [23] via the Raf-MEK-ERK pathway. Zhang et al. has demonstrated the expression of EGF and EGFR in chronic atrophic gastritis (CAG) and its significance in carcinogenesis. The expression levels of EGF in serum and urine from chronic atrophic gastritis patients were much higher than those in the control group. IL1B is critically involved in glycolysis and the induction of proinflammatory genes. IFNG is a proinflammatory factor, which regulates the expression of proinflammatory cytokine IFNG by CD4+ T cells under the condition of inflammation. The findings have important guiding significance for further understanding the dynamic regulation of human CD4+ T cell function in the inflammatory microenvironment. Prostaglandin endoperoxide synthases have 2 isoforms, PTGS1 and PTGS2, which are the rate-limiting enzymes in converting arachidonic acid into prostaglandins. PTGS1 is expressed constitutively, while PTGS2 is induced by cytokines and growth factors and is upregulated during inflammation. As we all know, PTGS2 (cyclooxygenase-2) is suppressed by aspirin, a nonsteroidal anti-inflammatory drug. PTGS2 can also mediate cell proliferation, angiogenesis, apoptosis, invasion, and immunosuppression to increase tumor progression [24]. The p53 protein is the product of TP53, a pivotal tumor suppressor gene, whose inactivation can result in gastric carcinogenesis [25, 26]. CCL2/MCP1 is an important indicator of an enhanced immune response [27–29]. Our network pharmacology research demonstrated that the active ingredients could effectively regulate the above critical targets of CAG.

The results of enrichment analysis revealed that the main pathways involved in the treatment process are HIF-1 signaling pathways, pathways of neurodegeneration-multiple disease, MAPK and PI3K-Akt signaling pathways, etc. HIF-1 is composed of an oxygen sensitive subunit HIF-1*α* and an aryl hydrocarbon nuclear translocator HIF-1*β*. It has also been shown to regulate the expression of VEGF, EPO, and glycolytic enzymes [30]. The Huazhuo Jiedu Hewei recipe could prevent and treat precancerous lesions of gastric cancer of CAG rats via downregulating the expression of HIF-1alpha and VEGF [31]. Huang et al. found that the abnormal expression of MAPK, a serine/threonine protein kinase, can accelerate the process of CAG [32]. Moreover, some research found that *Helicobacter pylori* infection could activate the PI3K/Akt pathway to promote the occurrence and development of CAG and tumors [33].

## 5. Conclusions

In summary, we identified 110 drug components and 26 potential targets in MLD by using network pharmacological methods. It was preliminarily predicted that MLD could regulate the targets of CCL2, CXCL8, EGF, PTGS2, IL1B, TP53, and IFNG through quercetin, kaempferol, and naringenin. The regulation of HIF-1 signaling pathways, pathways of neurodegeneration-multiple disease, MAPK, and PI3K-Akt signaling pathways potentially inhibit the inflammatory response, immune function, and cell apoptosis to treat CAG. Molecular docking demonstrated that the binding energy of quercetin with CCL2, CXCL8, EGF, PTGS2, IL1B, TP53, and IFNG is better than that of Omeprazole, a western drug used in clinical treatment of CAG. However, the experiments still need to verify the finding results in vivo and in vitro, such as the phosphorylation and expression of target gene, as well as the activity regulation of target protein.

## Figures and Tables

**Table 1:** Molecular docking results.

Molecule name	Formula	Binding free energy/(KJ.mol-1)						
		CCL2 1dok	CXCL8 5D14	EGF 1M17	IFNG 1hig	IL1B 4i1b	PTGS2 5IKR	TP53 3DCY
Quercetin	C15H10O7	−6.4	−6.6	−6.6	−7.3	−7.5	−9.2	−7.3
Omeprazole	C17H19N3 O3S	−5.7	−5.9	−6.4	−6.9	−6.7	−8.5	−6.6

## Data Availability

The datasets used and/or analyzed during the current study are available from the corresponding author on reasonable request.

## Supplementary Materials

Supplementary Materials Supplementary material 1: Supplementary Table S1. Potential targets information of MLD. Supplementary material 2: Supplementary Table S2. Potential targets information of CAG. Supplementary material 3: Supplementary Table S3. Ingredients of MLD for CAG treatment. Supplementary material 4: Supplementary Table S4. Targets of MLD for CAG treatment.
