# Supplementary material for: Network Pharmacology Approach to Investigate the Mechanism of Modified Liu Jun Zi Decoction in the Treatment of Chronic Atrophic Gastritis
Source: Evid Based Complement Alternat Med. 2022 Jun 17;2022:7536042. doi: 10.1155/2022/7536042 (PMC9232340; doi:10.1155/2022/7536042)
Supplement: Supplementary Materials — Supplementary material 1: Supplementary Table S1. Potential targets information of MLD. Supplementary material 2: Supplementary Table S2. Potential targets information of CAG. Supplementary material 3: Supplementary Table S3. Ingredients of MLD for CAG treatment. Supplementary material 4: Supplementary Table S4. Targets of MLD for CAG treatment. [file 7536042.f1.zip › 7536042.f1/Supplementary Table S1.pdf]

Drug	MolId	MolName	Gene Symbol
Baizhu	MOL000022	14-acetyl-	PTGS2
Baizhu	MOL000033	(3S,8S,9S,	PGR
Baizhu	MOL000049	3氻-aceto›	CHRM3
Baizhu	MOL000049	3氻-aceto›	CHRM1
Baizhu	MOL000049	3氻-aceto›	AR
Baizhu	MOL000049	3氻-aceto›	SCN5A
Baizhu	MOL000049	3氻-aceto›	PTGS2
Baizhu	MOL000049	3氻-aceto›	RXRA
Baizhu	MOL000049	3氻-aceto›	ACHE
Baizhu	MOL000049	3氻-aceto›	ADRA1A
Baizhu	MOL000049	3氻-aceto›	CHRM2
Baizhu	MOL000049	3氻-aceto›	ADRB2
Baizhu	MOL000049	3氻-aceto›	OPRM1
Baizhu	MOL000049	3氻-aceto›	GABRA1
Baizhu	MOL000049	3氻-aceto›	DPP4
Baizhu	MOL000072	8氻-ethox	PTGS2
Baizhu	MOL000072	8氻-ethox	GABRA1
Baizhu	MOL000072	8氻-ethox	NCOA2
Baizhu	MOL000072	8氻-ethox	NCOA1
Banxia	MOL001755	24-Ethylcl	PGR
Banxia	MOL001755	24-Ethylcl	NR3C2
Banxia	MOL002670	Cavidine	PTGS1
Banxia	MOL002670	Cavidine	CHRM3
Banxia	MOL002670	Cavidine	KCNH2
Banxia	MOL002670	Cavidine	CHRM1
Banxia	MOL002670	Cavidine	ADRB1
Banxia	MOL002670	Cavidine	SCN5A
Banxia	MOL002670	Cavidine	CHRM5
Banxia	MOL002670	Cavidine	PTGS2
Banxia	MOL002670	Cavidine	HTR3A
Banxia	MOL002670	Cavidine	ADRA2C
Banxia	MOL002670	Cavidine	CHRM4
Banxia	MOL002670	Cavidine	RXRA
Banxia	MOL002670	Cavidine	OPRD1
Banxia	MOL002670	Cavidine	ADRA1B
Banxia	MOL002670	Cavidine	ADRB2
Banxia	MOL002670	Cavidine	ADRA1D
Banxia	MOL002670	Cavidine	OPRM1
Banxia	MOL002670	Cavidine	HSP90AB1
Banxia	MOL002670	Cavidine	RXRB
Banxia	MOL002670	Cavidine	PCP4
Banxia	MOL002670	Cavidine	DRD1
Banxia	MOL002670	Cavidine	SLC6A4
Banxia	MOL002670	Cavidine	F7
Banxia	MOL002670	Cavidine	PDE10A
Banxia	MOL002714	baicalein	PTGS1
Banxia	MOL002714	baicalein	AR
Banxia	MOL002714	baicalein	PTGS2
Banxia	MOL002714	baicalein	HSP90AB1
Banxia	MOL002714	baicalein	DPP4

Banxia	MOL002714	baicalein	PRSS1
Banxia	MOL002714	baicalein	NCOA2
Banxia	MOL002714	baicalein	NCOA1
Banxia	MOL002714	baicalein	PCP4
Banxia	MOL002714	baicalein	RELA
Banxia	MOL002714	baicalein	AKT1
Banxia	MOL002714	baicalein	VEGFA
Banxia	MOL002714	baicalein	BCL2
Banxia	MOL002714	baicalein	FOS
Banxia	MOL002714	baicalein	BAX
Banxia	MOL002714	baicalein	MMP9
Banxia	MOL002714	baicalein	CASP3
Banxia	MOL002714	baicalein	TP53
Banxia	MOL002714	baicalein	HIF1A
Banxia	MOL002714	baicalein	FOSL1
Banxia	MOL002714	baicalein	FOSL2
Banxia	MOL002714	baicalein	CDK1
Banxia	MOL002714	baicalein	CCNB1
Banxia	MOL002714	baicalein	MPO
Banxia	MOL002714	baicalein	AHR
Banxia	MOL002714	baicalein	IGF2
Banxia	MOL002714	baicalein	CYCS
Banxia	MOL002714	baicalein	NFATC1
Banxia	MOL002714	baicalein	TDRD7
Banxia	MOL002714	baicalein	EGLN1
Banxia	MOL002714	baicalein	NOX5
Banxia	MOL002714	baicalein	FABP5
Banxia	MOL002714	baicalein	APOD
Banxia	MOL000358	beta-sitos	PGR
Banxia	MOL000358	beta-sitos	NCOA2
Banxia	MOL000358	beta-sitos	PTGS1
Banxia	MOL000358	beta-sitos	PTGS2
Banxia	MOL000358	beta-sitos	HSP90AB1
Banxia	MOL000358	beta-sitos	KCNH2
Banxia	MOL000358	beta-sitos	DRD1
Banxia	MOL000358	beta-sitos	CHRM3
Banxia	MOL000358	beta-sitos	CHRM1
Banxia	MOL000358	beta-sitos	SCN5A
Banxia	MOL000358	beta-sitos	CHRM4
Banxia	MOL000358	beta-sitos	ADRA1A
Banxia	MOL000358	beta-sitos	CHRM2
Banxia	MOL000358	beta-sitos	ADRA1B
Banxia	MOL000358	beta-sitos	ADRB2
Banxia	MOL000358	beta-sitos	CHRNA2
Banxia	MOL000358	beta-sitos	SLC6A4
Banxia	MOL000358	beta-sitos	OPRM1
Banxia	MOL000358	beta-sitos	GABRA1
Banxia	MOL000358	beta-sitos	BCL2
Banxia	MOL000358	beta-sitos	BAX
Banxia	MOL000358	beta-sitos	CASP9
Banxia	MOL000358	beta-sitos	JUN

Banxia	MOL000358	beta-sitos	CASP3
Banxia	MOL000358	beta-sitos	CASP8
Banxia	MOL000358	beta-sitos	PRKCA
Banxia	MOL000358	beta-sitos	PON1
Banxia	MOL000358	beta-sitos	MAP2
Banxia	MOL000449	Stigmaster	PGR
Banxia	MOL000449	Stigmaster	NR3C2
Banxia	MOL000449	Stigmaster	NCOA2
Banxia	MOL000449	Stigmaster	ADH1C
Banxia	MOL000449	Stigmaster	IGHG1
Banxia	MOL000449	Stigmaster	RXRA
Banxia	MOL000449	Stigmaster	NCOA1
Banxia	MOL000449	Stigmaster	PTGS1
Banxia	MOL000449	Stigmaster	PTGS2
Banxia	MOL000449	Stigmaster	ADRA2A
Banxia	MOL000449	Stigmaster	SLC6A2
Banxia	MOL000449	Stigmaster	SLC6A3
Banxia	MOL000449	Stigmaster	ADRB2
Banxia	MOL000449	Stigmaster	AKR1B1
Banxia	MOL000449	Stigmaster	PLAU
Banxia	MOL000449	Stigmaster	LTA4H
Banxia	MOL000449	Stigmaster	MAOB
Banxia	MOL000449	Stigmaster	MAOA
Banxia	MOL000449	Stigmaster	CTRB1
Banxia	MOL000449	Stigmaster	CHRM3
Banxia	MOL000449	Stigmaster	CHRM1
Banxia	MOL000449	Stigmaster	ADRB1
Banxia	MOL000449	Stigmaster	SCN5A
Banxia	MOL000449	Stigmaster	ADRA1A
Banxia	MOL000449	Stigmaster	CHRM2
Banxia	MOL000449	Stigmaster	ADRA1B
Banxia	MOL000449	Stigmaster	GABRA1
Banxia	MOL005030	gondoic ac	PTGS1
Banxia	MOL005030	gondoic ac	NCOA2
Banxia	MOL000519	coniferin	CHRM3
Banxia	MOL000519	coniferin	CHRM1
Banxia	MOL000519	coniferin	ESR1
Banxia	MOL000519	coniferin	AR
Banxia	MOL000519	coniferin	SCN5A
Banxia	MOL000519	coniferin	PPARG
Banxia	MOL000519	coniferin	PTGS2
Banxia	MOL000519	coniferin	CA2
Banxia	MOL000519	coniferin	ADRA1B
Banxia	MOL000519	coniferin	ADRB2
Banxia	MOL000519	coniferin	ADRA1D
Banxia	MOL000519	coniferin	OPRM1
Banxia	MOL000519	coniferin	CDK2
Banxia	MOL000519	coniferin	DPEP1
Banxia	MOL000519	coniferin	IGHG1
Banxia	MOL000519	coniferin	CCNA2
Banxia	MOL000519	coniferin	NCOA2

Banxia	MOL000519	coniferin	NCOA1
Banxia	MOL006936	10,13-eicc	PTGS1
Banxia	MOL006936	10,13-eicc	NCOA2
Banxia	MOL006957	(3S,6S)-3-	AR
Banxia	MOL006957	(3S,6S)-3-	PTGS2
Banxia	MOL006957	(3S,6S)-3-	ADRB2
Banxia	MOL006957	(3S,6S)-3-	PCP4
Banxia	MOL003578	Cycloarter	NR3C2
Banxia	MOL006967	beta-D-Rit	PNP
Banxia	MOL006967	beta-D-Rit	PTGS2
Chenpi	MOL000359	sitosterol	PGR
Chenpi	MOL000359	sitosterol	NCOA2
Chenpi	MOL000359	sitosterol	NR3C2
Chenpi	MOL004328	naringenir	PTGS1
Chenpi	MOL004328	naringenir	ESR1
Chenpi	MOL004328	naringenir	PTGS2
Chenpi	MOL004328	naringenir	HSP90AB1
Chenpi	MOL004328	naringenir	DPEP1
Chenpi	MOL004328	naringenir	RELA
Chenpi	MOL004328	naringenir	AKT1
Chenpi	MOL004328	naringenir	BCL2
Chenpi	MOL004328	naringenir	MAPK3
Chenpi	MOL004328	naringenir	MAPK1
Chenpi	MOL004328	naringenir	CASP3
Chenpi	MOL004328	naringenir	FASN
Chenpi	MOL004328	naringenir	LDLR
Chenpi	MOL004328	naringenir	BAD
Chenpi	MOL004328	naringenir	SOD1
Chenpi	MOL004328	naringenir	CAT
Chenpi	MOL004328	naringenir	PPARG
Chenpi	MOL004328	naringenir	MTTP
Chenpi	MOL004328	naringenir	APOB
Chenpi	MOL004328	naringenir	PLB1
Chenpi	MOL004328	naringenir	HMGCR
Chenpi	MOL004328	naringenir	CYP19A1
Chenpi	MOL004328	naringenir	GSTP1
Chenpi	MOL004328	naringenir	UGT1A1
Chenpi	MOL004328	naringenir	PPARA
Chenpi	MOL004328	naringenir	SREBF1
Chenpi	MOL004328	naringenir	GSR
Chenpi	MOL004328	naringenir	ABCC1
Chenpi	MOL004328	naringenir	ADIPOR2
Chenpi	MOL004328	naringenir	SOAT2
Chenpi	MOL004328	naringenir	AKR1C1
Chenpi	MOL004328	naringenir	GOT1
Chenpi	MOL004328	naringenir	ABAT
Chenpi	MOL004328	naringenir	CES1
Chenpi	MOL004328	naringenir	SOAT1
Chenpi	MOL005100	5,7-dihydr	PTGS1
Chenpi	MOL005100	5,7-dihydr	PTGS2
Chenpi	MOL005100	5,7-dihydr	HSP90AB1

Chenpi	MOL005100	5,7-dihydr	PCP4
Chenpi	MOL005100	5,7-dihydr	SCN5A
Chenpi	MOL005100	5,7-dihydr	NCOA2
Chenpi	MOL005100	5,7-dihydr	NCOA1
Chenpi	MOL005815	Citromitir	KCNH2
Chenpi	MOL005815	Citromitir	SCN5A
Chenpi	MOL005815	Citromitir	PTGS2
Chenpi	MOL005815	Citromitir	F7
Chenpi	MOL005815	Citromitir	HSP90AB1
Chenpi	MOL005815	Citromitir	NCOA2
Chenpi	MOL005815	Citromitir	PCP4
Chenpi	MOL005828	nobiletin	NOS2
Chenpi	MOL005828	nobiletin	PTGS1
Chenpi	MOL005828	nobiletin	KCNH2
Chenpi	MOL005828	nobiletin	ESR1
Chenpi	MOL005828	nobiletin	AR
Chenpi	MOL005828	nobiletin	PPARG
Chenpi	MOL005828	nobiletin	PTGS2
Chenpi	MOL005828	nobiletin	F7
Chenpi	MOL005828	nobiletin	ESR2
Chenpi	MOL005828	nobiletin	DPP4
Chenpi	MOL005828	nobiletin	HSP90AB1
Chenpi	MOL005828	nobiletin	CHEK1
Chenpi	MOL005828	nobiletin	PRSS1
Chenpi	MOL005828	nobiletin	NCOA2
Chenpi	MOL005828	nobiletin	PCP4
Chenpi	MOL005828	nobiletin	GSK3B
Chenpi	MOL005828	nobiletin	SCN5A
Chenpi	MOL005828	nobiletin	BCL2
Chenpi	MOL005828	nobiletin	BAX
Chenpi	MOL005828	nobiletin	CASP9
Chenpi	MOL005828	nobiletin	MMP9
Chenpi	MOL005828	nobiletin	JUN
Chenpi	MOL005828	nobiletin	TP53
Chenpi	MOL005828	nobiletin	MAPK8
Chenpi	MOL005828	nobiletin	TIMP1
Chenpi	MOL005828	nobiletin	PPARG
Chenpi	MOL005828	nobiletin	CREB1
Chenpi	MOL005828	nobiletin	PLA2G4A
Chenpi	MOL005828	nobiletin	CD163
Chenpi	MOL005828	nobiletin	EPHB2
Fuling	MOL000273	(2R)-2-[(	NR3C2
Fuling	MOL000273	(2R)-2-[(	NCOA2
Fuling	MOL000275	trametenol	NR3C2
Fuling	MOL000279	Cerevister	NR3C2
Fuling	MOL000282	ergosta-7,	PGR
Fuling	MOL000283	Ergosterol	PGR
Fuling	MOL000296	hederageni	PGR
Fuling	MOL000296	hederageni	NCOA2
Fuling	MOL000296	hederageni	CHRM3
Fuling	MOL000296	hederageni	CHRM1

Fuling	MOL000296	hederageni	CHRM2
Fuling	MOL000296	hederageni	ADRA1B
Fuling	MOL000296	hederageni	GABRA1
Fuling	MOL000296	hederageni	GRIA2
Fuling	MOL000296	hederageni	IGHG1
Fuling	MOL000296	hederageni	ADH1B
Fuling	MOL000296	hederageni	ADH1C
Fuling	MOL000296	hederageni	LYZ
Fuling	MOL000296	hederageni	PTGS1
Fuling	MOL000296	hederageni	SCN5A
Fuling	MOL000296	hederageni	PTGS2
Fuling	MOL000296	hederageni	RXRA
Fuling	MOL000296	hederageni	SLC6A2
Gancao	MOL001484	Inermine	PTGS1
Gancao	MOL001484	Inermine	CHRM3
Gancao	MOL001484	Inermine	SCN5A
Gancao	MOL001484	Inermine	PTGS2
Gancao	MOL001484	Inermine	HTR3A
Gancao	MOL001484	Inermine	RXRA
Gancao	MOL001484	Inermine	ADRA1B
Gancao	MOL001484	Inermine	ADRA1D
Gancao	MOL001484	Inermine	IGHG1
Gancao	MOL001484	Inermine	PRSS1
Gancao	MOL001484	Inermine	PCP4
Gancao	MOL001484	Inermine	CHRM1
Gancao	MOL001484	Inermine	ADRB2
Gancao	MOL001484	Inermine	OPRM1
Gancao	MOL001484	Inermine	HSP90AB1
Gancao	MOL001792	DFV	PTGS1
Gancao	MOL001792	DFV	ESR1
Gancao	MOL001792	DFV	PTGS2
Gancao	MOL001792	DFV	RXRA
Gancao	MOL001792	DFV	ADRB2
Gancao	MOL001792	DFV	HSP90AB1
Gancao	MOL001792	DFV	DPEP1
Gancao	MOL001792	DFV	MAOB
Gancao	MOL001792	DFV	SLC6A4
Gancao	MOL001792	DFV	PKIA
Gancao	MOL000211	Mairin	PGR
Gancao	MOL002311	Glycyrol	NOS2
Gancao	MOL002311	Glycyrol	ESR1
Gancao	MOL002311	Glycyrol	PPARG
Gancao	MOL002311	Glycyrol	PTGS2
Gancao	MOL002311	Glycyrol	KDR
Gancao	MOL002311	Glycyrol	MAPK14
Gancao	MOL002311	Glycyrol	GSK3B
Gancao	MOL002311	Glycyrol	CHEK1
Gancao	MOL002311	Glycyrol	CCNA2
Gancao	MOL000239	Jaranol	NOS2
Gancao	MOL000239	Jaranol	PTGS1
Gancao	MOL000239	Jaranol	AR

Gancao	MOL000239	Jaranol	SCN5A
Gancao	MOL000239	Jaranol	PTGS2
Gancao	MOL000239	Jaranol	ESR2
Gancao	MOL000239	Jaranol	DPP4
Gancao	MOL000239	Jaranol	HSP90AB1
Gancao	MOL000239	Jaranol	CDK2
Gancao	MOL000239	Jaranol	CHEK1
Gancao	MOL000239	Jaranol	PRSS1
Gancao	MOL000239	Jaranol	NCOA2
Gancao	MOL000239	Jaranol	PCP4
Gancao	MOL002565	Medicarpir	NOS2
Gancao	MOL002565	Medicarpir	PTGS1
Gancao	MOL002565	Medicarpir	DRD1
Gancao	MOL002565	Medicarpir	CHRM3
Gancao	MOL002565	Medicarpir	CHRM1
Gancao	MOL002565	Medicarpir	ESR1
Gancao	MOL002565	Medicarpir	SCN5A
Gancao	MOL002565	Medicarpir	CHRM5
Gancao	MOL002565	Medicarpir	PTGS2
Gancao	MOL002565	Medicarpir	CHRM4
Gancao	MOL002565	Medicarpir	RXRA
Gancao	MOL002565	Medicarpir	ADRA1A
Gancao	MOL002565	Medicarpir	CHRM2
Gancao	MOL002565	Medicarpir	ADRA1B
Gancao	MOL002565	Medicarpir	SLC6A3
Gancao	MOL002565	Medicarpir	ADRB2
Gancao	MOL002565	Medicarpir	SLC6A4
Gancao	MOL002565	Medicarpir	OPRM1
Gancao	MOL002565	Medicarpir	ESR2
Gancao	MOL002565	Medicarpir	DPP4
Gancao	MOL002565	Medicarpir	MAPK10
Gancao	MOL002565	Medicarpir	HSP90AB1
Gancao	MOL002565	Medicarpir	CDK2
Gancao	MOL002565	Medicarpir	PRSS1
Gancao	MOL002565	Medicarpir	CCNA2
Gancao	MOL002565	Medicarpir	PCP4
Gancao	MOL002565	Medicarpir	OPRD1
Gancao	MOL002565	Medicarpir	ADRA1D
Gancao	MOL000354	isorhamnet	NOS2
Gancao	MOL000354	isorhamnet	PTGS1
Gancao	MOL000354	isorhamnet	ESR1
Gancao	MOL000354	isorhamnet	AR
Gancao	MOL000354	isorhamnet	PPARG
Gancao	MOL000354	isorhamnet	PTGS2
Gancao	MOL000354	isorhamnet	ESR2
Gancao	MOL000354	isorhamnet	DPP4
Gancao	MOL000354	isorhamnet	MAPK14
Gancao	MOL000354	isorhamnet	GSK3B
Gancao	MOL000354	isorhamnet	HSP90AB1
Gancao	MOL000354	isorhamnet	CDK2
Gancao	MOL000354	isorhamnet	PRSS1

Gancao	MOL000354	isorhamnet	CCNA2
Gancao	MOL000354	isorhamnet	NCOA2
Gancao	MOL000354	isorhamnet	PCP4
Gancao	MOL000354	isorhamnet	PYGM
Gancao	MOL000354	isorhamnet	PPARD
Gancao	MOL000354	isorhamnet	CHEK1
Gancao	MOL000354	isorhamnet	AKR1B1
Gancao	MOL000354	isorhamnet	NCOA1
Gancao	MOL000354	isorhamnet	F7
Gancao	MOL000354	isorhamnet	ACHE
Gancao	MOL000354	isorhamnet	GABRA1
Gancao	MOL000354	isorhamnet	MAOB
Gancao	MOL000354	isorhamnet	GRIA2
Gancao	MOL000354	isorhamnet	RELA
Gancao	MOL000354	isorhamnet	NCF1
Gancao	MOL000354	isorhamnet	OLR1
Gancao	MOL000359	sitosterol	PGR
Gancao	MOL000359	sitosterol	NCOA2
Gancao	MOL000359	sitosterol	NR3C2
Gancao	MOL003656	Lupiwighte	NOS2
Gancao	MOL003656	Lupiwighte	ESR1
Gancao	MOL003656	Lupiwighte	AR
Gancao	MOL003656	Lupiwighte	SCN5A
Gancao	MOL003656	Lupiwighte	PPARG
Gancao	MOL003656	Lupiwighte	PTGS2
Gancao	MOL003656	Lupiwighte	ESR2
Gancao	MOL003656	Lupiwighte	DPP4
Gancao	MOL003656	Lupiwighte	MAPK14
Gancao	MOL003656	Lupiwighte	GSK3B
Gancao	MOL003656	Lupiwighte	HSP90AB1
Gancao	MOL003656	Lupiwighte	CDK2
Gancao	MOL003656	Lupiwighte	CHEK1
Gancao	MOL003656	Lupiwighte	PRSS1
Gancao	MOL003656	Lupiwighte	CCNA2
Gancao	MOL003656	Lupiwighte	NCOA2
Gancao	MOL003656	Lupiwighte	PCP4
Gancao	MOL003896	7-Methoxy-	NOS2
Gancao	MOL003896	7-Methoxy-	PTGS1
Gancao	MOL003896	7-Methoxy-	DRD1
Gancao	MOL003896	7-Methoxy-	CHRM3
Gancao	MOL003896	7-Methoxy-	CHRM1
Gancao	MOL003896	7-Methoxy-	ESR1
Gancao	MOL003896	7-Methoxy-	AR
Gancao	MOL003896	7-Methoxy-	ADRB1
Gancao	MOL003896	7-Methoxy-	SCN5A
Gancao	MOL003896	7-Methoxy-	PPARG
Gancao	MOL003896	7-Methoxy-	PTGS2
Gancao	MOL003896	7-Methoxy-	RXRA
Gancao	MOL003896	7-Methoxy-	ACHE
Gancao	MOL003896	7-Methoxy-	ADRA1B
Gancao	MOL003896	7-Methoxy-	SLC6A3

Gancao	MOL003896	7-Methoxy-ADRB2
Gancao	MOL003896	7-Methoxy-ADRA1D
Gancao	MOL003896	7-Methoxy-SLC6A4
Gancao	MOL003896	7-Methoxy-ESR2
Gancao	MOL003896	7-Methoxy-GABRA1
Gancao	MOL003896	7-Methoxy-DPP4
Gancao	MOL003896	7-Methoxy-MAPK14
Gancao	MOL003896	7-Methoxy-GSK3B
Gancao	MOL003896	7-Methoxy-HSP90AB1
Gancao	MOL003896	7-Methoxy-CDK2
Gancao	MOL003896	7-Methoxy-LTA4H
Gancao	MOL003896	7-Methoxy-MAOB
Gancao	MOL003896	7-Methoxy-CHEK1
Gancao	MOL003896	7-Methoxy-IGHG1
Gancao	MOL003896	7-Methoxy-PRSS1
Gancao	MOL003896	7-Methoxy-CCNA2
Gancao	MOL003896	7-Methoxy-NCOA1
Gancao	MOL003896	7-Methoxy-PKIA
Gancao	MOL003896	7-Methoxy-PCP4
Gancao	MOL003896	7-Methoxy-CHRM5
Gancao	MOL003896	7-Methoxy-OPRM1
Gancao	MOL003896	7-Methoxy-NCOA2
Gancao	MOL000392	formononetNOS2
Gancao	MOL000392	formononetPTGS1
Gancao	MOL000392	formononetCHRM1
Gancao	MOL000392	formononetESR1
Gancao	MOL000392	formononetAR
Gancao	MOL000392	formononetPPARG
Gancao	MOL000392	formononetPTGS2
Gancao	MOL000392	formononetRXRA
Gancao	MOL000392	formononetADRA1A
Gancao	MOL000392	formononetSLC6A3
Gancao	MOL000392	formononetADRB2
Gancao	MOL000392	formononetSLC6A4
Gancao	MOL000392	formononetESR2
Gancao	MOL000392	formononetDPP4
Gancao	MOL000392	formononetMAPK14
Gancao	MOL000392	formononetGSK3B
Gancao	MOL000392	formononetHSP90AB1
Gancao	MOL000392	formononetCDK2
Gancao	MOL000392	formononetMAOB
Gancao	MOL000392	formononetCHEK1
Gancao	MOL000392	formononetPRSS1
Gancao	MOL000392	formononetCCNA2
Gancao	MOL000392	formononetPCP4
Gancao	MOL000392	formononetPKIA
Gancao	MOL000392	formononetACHE
Gancao	MOL000392	formononetDPEP1
Gancao	MOL000392	formononetJUN
Gancao	MOL000392	formononetPPARG
Gancao	MOL000392	formononetIL4

Gancao	MOL000392	formononet	ATP5F1B
Gancao	MOL000392	formononet	HSD3B2
Gancao	MOL000392	formononet	HSD3B1
Gancao	MOL000417	Calycosin	NOS2
Gancao	MOL000417	Calycosin	PTGS1
Gancao	MOL000417	Calycosin	ESR1
Gancao	MOL000417	Calycosin	AR
Gancao	MOL000417	Calycosin	PPARG
Gancao	MOL000417	Calycosin	PTGS2
Gancao	MOL000417	Calycosin	RXRA
Gancao	MOL000417	Calycosin	ESR2
Gancao	MOL000417	Calycosin	DPP4
Gancao	MOL000417	Calycosin	MAPK14
Gancao	MOL000417	Calycosin	GSK3B
Gancao	MOL000417	Calycosin	HSP90AB1
Gancao	MOL000417	Calycosin	CDK2
Gancao	MOL000417	Calycosin	CHEK1
Gancao	MOL000417	Calycosin	PRSS1
Gancao	MOL000417	Calycosin	CCNA2
Gancao	MOL000417	Calycosin	NCOA2
Gancao	MOL000417	Calycosin	PCP4
Gancao	MOL000417	Calycosin	ADRB2
Gancao	MOL000422	kaempferol	NOS2
Gancao	MOL000422	kaempferol	PTGS1
Gancao	MOL000422	kaempferol	AR
Gancao	MOL000422	kaempferol	PPARG
Gancao	MOL000422	kaempferol	PTGS2
Gancao	MOL000422	kaempferol	HSP90AB1
Gancao	MOL000422	kaempferol	NCOA2
Gancao	MOL000422	kaempferol	DPP4
Gancao	MOL000422	kaempferol	PRSS1
Gancao	MOL000422	kaempferol	PGR
Gancao	MOL000422	kaempferol	CHRM1
Gancao	MOL000422	kaempferol	ACHE
Gancao	MOL000422	kaempferol	SLC6A2
Gancao	MOL000422	kaempferol	CHRM2
Gancao	MOL000422	kaempferol	ADRA1B
Gancao	MOL000422	kaempferol	GABRA1
Gancao	MOL000422	kaempferol	F7
Gancao	MOL000422	kaempferol	PCP4
Gancao	MOL000422	kaempferol	RELA
Gancao	MOL000422	kaempferol	IKBKB
Gancao	MOL000422	kaempferol	AKT1
Gancao	MOL000422	kaempferol	BCL2
Gancao	MOL000422	kaempferol	BAX
Gancao	MOL000422	kaempferol	TNFAIP6
Gancao	MOL000422	kaempferol	JUN
Gancao	MOL000422	kaempferol	AHSA1
Gancao	MOL000422	kaempferol	CASP3
Gancao	MOL000422	kaempferol	MAPK8
Gancao	MOL000422	kaempferol	MMP1

Gancao	MOL000422	kaempferolSTAT1
Gancao	MOL000422	kaempferolCDK1
Gancao	MOL000422	kaempferolPPARG
Gancao	MOL000422	kaempferolHMOX1
Gancao	MOL000422	kaempferolCYP3A4
Gancao	MOL000422	kaempferolCYP1A2
Gancao	MOL000422	kaempferolCYP1A1
Gancao	MOL000422	kaempferolICAM1
Gancao	MOL000422	kaempferolSELE
Gancao	MOL000422	kaempferolVCAM1
Gancao	MOL000422	kaempferolNR1I2
Gancao	MOL000422	kaempferolCYP1B1
Gancao	MOL000422	kaempferolALOX5
Gancao	MOL000422	kaempferolHAS2
Gancao	MOL000422	kaempferolGSTP1
Gancao	MOL000422	kaempferolAHR
Gancao	MOL000422	kaempferolPSMD3
Gancao	MOL000422	kaempferolSLC2A4
Gancao	MOL000422	kaempferolNR1I3
Gancao	MOL000422	kaempferolINSRR
Gancao	MOL000422	kaempferolDIO1
Gancao	MOL000422	kaempferolPPP3CA
Gancao	MOL000422	kaempferolGSTM1
Gancao	MOL000422	kaempferolGSTM2
Gancao	MOL000422	kaempferolAKR1C3
Gancao	MOL000422	kaempferolSLPI
Gancao	MOL004328	naringenirPTGS1
Gancao	MOL004328	naringenirESR1
Gancao	MOL004328	naringenirPTGS2
Gancao	MOL004328	naringenirHSP90AB1
Gancao	MOL004328	naringenirDPEP1
Gancao	MOL004328	naringenirRELA
Gancao	MOL004328	naringenirAKT1
Gancao	MOL004328	naringenirBCL2
Gancao	MOL004328	naringenirMAPK3
Gancao	MOL004328	naringenirMAPK1
Gancao	MOL004328	naringenirCASP3
Gancao	MOL004328	naringenirFASN
Gancao	MOL004328	naringenirLDLR
Gancao	MOL004328	naringenirBAD
Gancao	MOL004328	naringenirSOD1
Gancao	MOL004328	naringenirCAT
Gancao	MOL004328	naringenirPPARG
Gancao	MOL004328	naringenirMTTP
Gancao	MOL004328	naringenirAPOB
Gancao	MOL004328	naringenirPLB1
Gancao	MOL004328	naringenirHMGCR
Gancao	MOL004328	naringenirCYP19A1
Gancao	MOL004328	naringenirGSTP1
Gancao	MOL004328	naringenirUGT1A1
Gancao	MOL004328	naringenirPPARA

Gancao	MOL004328	naringenir	SREBF1
Gancao	MOL004328	naringenir	GSR
Gancao	MOL004328	naringenir	ABCC1
Gancao	MOL004328	naringenir	ADIPOR2
Gancao	MOL004328	naringenir	SOAT2
Gancao	MOL004328	naringenir	AKR1C1
Gancao	MOL004328	naringenir	GOT1
Gancao	MOL004328	naringenir	ABAT
Gancao	MOL004328	naringenir	CES1
Gancao	MOL004328	naringenir	SOAT1
Gancao	MOL004805	(2S)-2-[4-	NOS2
Gancao	MOL004805	(2S)-2-[4-	KCNH2
Gancao	MOL004805	(2S)-2-[4-	ESR1
Gancao	MOL004805	(2S)-2-[4-	AR
Gancao	MOL004805	(2S)-2-[4-	PPARG
Gancao	MOL004805	(2S)-2-[4-	PTGS2
Gancao	MOL004805	(2S)-2-[4-	ESR2
Gancao	MOL004805	(2S)-2-[4-	MAPK14
Gancao	MOL004805	(2S)-2-[4-	GSK3B
Gancao	MOL004805	(2S)-2-[4-	PCP4
Gancao	MOL004806	euchrenone	NOS2
Gancao	MOL004806	euchrenone	KCNH2
Gancao	MOL004806	euchrenone	ESR1
Gancao	MOL004806	euchrenone	SCN5A
Gancao	MOL004806	euchrenone	PTGS2
Gancao	MOL004806	euchrenone	ESR2
Gancao	MOL004806	euchrenone	BACE2
Gancao	MOL004806	euchrenone	PCP4
Gancao	MOL004808	glyasperir	NOS2
Gancao	MOL004808	glyasperir	ESR1
Gancao	MOL004808	glyasperir	AR
Gancao	MOL004808	glyasperir	PPARG
Gancao	MOL004808	glyasperir	PTGS2
Gancao	MOL004808	glyasperir	F7
Gancao	MOL004808	glyasperir	KDR
Gancao	MOL004808	glyasperir	ACHE
Gancao	MOL004808	glyasperir	ESR2
Gancao	MOL004808	glyasperir	DPP4
Gancao	MOL004808	glyasperir	GSK3B
Gancao	MOL004808	glyasperir	HSP90AB1
Gancao	MOL004808	glyasperir	CDK2
Gancao	MOL004808	glyasperir	PRSS1
Gancao	MOL004808	glyasperir	CCNA2
Gancao	MOL004808	glyasperir	NCOA2
Gancao	MOL004808	glyasperir	PCP4
Gancao	MOL004810	glyasperir	NOS2
Gancao	MOL004810	glyasperir	PTGS1
Gancao	MOL004810	glyasperir	ESR1
Gancao	MOL004810	glyasperir	AR
Gancao	MOL004810	glyasperir	SCN5A
Gancao	MOL004810	glyasperir	PPARG

Gancao	MOL004810	glyasperir	PTGS2
Gancao	MOL004810	glyasperir	ESR2
Gancao	MOL004810	glyasperir	MAPK14
Gancao	MOL004810	glyasperir	GSK3B
Gancao	MOL004810	glyasperir	HSP90AB1
Gancao	MOL004810	glyasperir	CDK2
Gancao	MOL004810	glyasperir	PRSS1
Gancao	MOL004810	glyasperir	CCNA2
Gancao	MOL004810	glyasperir	PCP4
Gancao	MOL004811	Glyasperir	NOS2
Gancao	MOL004811	Glyasperir	KCNH2
Gancao	MOL004811	Glyasperir	ESR1
Gancao	MOL004811	Glyasperir	AR
Gancao	MOL004811	Glyasperir	SCN5A
Gancao	MOL004811	Glyasperir	PPARG
Gancao	MOL004811	Glyasperir	PTGS2
Gancao	MOL004811	Glyasperir	RXRA
Gancao	MOL004811	Glyasperir	ACHE
Gancao	MOL004811	Glyasperir	ESR2
Gancao	MOL004811	Glyasperir	DPP4
Gancao	MOL004811	Glyasperir	MAPK14
Gancao	MOL004811	Glyasperir	GSK3B
Gancao	MOL004811	Glyasperir	HSP90AB1
Gancao	MOL004811	Glyasperir	CDK2
Gancao	MOL004811	Glyasperir	CHEK1
Gancao	MOL004811	Glyasperir	PRSS1
Gancao	MOL004811	Glyasperir	CCNA2
Gancao	MOL004811	Glyasperir	NCOA2
Gancao	MOL004811	Glyasperir	PCP4
Gancao	MOL004814	Isotrifoli	NOS2
Gancao	MOL004814	Isotrifoli	ESR1
Gancao	MOL004814	Isotrifoli	AR
Gancao	MOL004814	Isotrifoli	PTGS2
Gancao	MOL004814	Isotrifoli	ESR2
Gancao	MOL004814	Isotrifoli	MAPK14
Gancao	MOL004814	Isotrifoli	GSK3B
Gancao	MOL004814	Isotrifoli	HSP90AB1
Gancao	MOL004814	Isotrifoli	CDK2
Gancao	MOL004814	Isotrifoli	CHEK1
Gancao	MOL004814	Isotrifoli	CCNA2
Gancao	MOL004815	(E)-1-(2,4	NOS2
Gancao	MOL004815	(E)-1-(2,4	PTGS1
Gancao	MOL004815	(E)-1-(2,4	ESR1
Gancao	MOL004815	(E)-1-(2,4	AR
Gancao	MOL004815	(E)-1-(2,4	SCN5A
Gancao	MOL004815	(E)-1-(2,4	PPARG
Gancao	MOL004815	(E)-1-(2,4	PTGS2
Gancao	MOL004815	(E)-1-(2,4	CA2
Gancao	MOL004815	(E)-1-(2,4	RXRA
Gancao	MOL004815	(E)-1-(2,4	ADRA1B
Gancao	MOL004815	(E)-1-(2,4	ESR2

Gancao	MOL004815	(E)-1-(2,4	MAPK14
Gancao	MOL004815	(E)-1-(2,4	GSK3B
Gancao	MOL004815	(E)-1-(2,4	CDK2
Gancao	MOL004815	(E)-1-(2,4	CHEK1
Gancao	MOL004815	(E)-1-(2,4	CCNA2
Gancao	MOL004815	(E)-1-(2,4	NCOA2
Gancao	MOL004815	(E)-1-(2,4	PCP4
Gancao	MOL004820	kanzonols	NOS2
Gancao	MOL004820	kanzonols	PTGS1
Gancao	MOL004820	kanzonols	ESR1
Gancao	MOL004820	kanzonols	AR
Gancao	MOL004820	kanzonols	SCN5A
Gancao	MOL004820	kanzonols	PPARG
Gancao	MOL004820	kanzonols	PTGS2
Gancao	MOL004820	kanzonols	RXRA
Gancao	MOL004820	kanzonols	ESR2
Gancao	MOL004820	kanzonols	MAPK14
Gancao	MOL004820	kanzonols	GSK3B
Gancao	MOL004820	kanzonols	CDK2
Gancao	MOL004820	kanzonols	CHEK1
Gancao	MOL004820	kanzonols	PRSS1
Gancao	MOL004820	kanzonols	CCNA2
Gancao	MOL004820	kanzonols	NCOA2
Gancao	MOL004820	kanzonols	NCOA1
Gancao	MOL004820	kanzonols	PCP4
Gancao	MOL004824	(2S)-6-(2,	NOS2
Gancao	MOL004824	(2S)-6-(2,	ESR1
Gancao	MOL004824	(2S)-6-(2,	AR
Gancao	MOL004824	(2S)-6-(2,	PPARG
Gancao	MOL004824	(2S)-6-(2,	PTGS2
Gancao	MOL004824	(2S)-6-(2,	F7
Gancao	MOL004824	(2S)-6-(2,	KDR
Gancao	MOL004824	(2S)-6-(2,	ACHE
Gancao	MOL004824	(2S)-6-(2,	ESR2
Gancao	MOL004824	(2S)-6-(2,	DPP4
Gancao	MOL004824	(2S)-6-(2,	MAPK14
Gancao	MOL004824	(2S)-6-(2,	GSK3B
Gancao	MOL004824	(2S)-6-(2,	CDK2
Gancao	MOL004824	(2S)-6-(2,	CHEK1
Gancao	MOL004824	(2S)-6-(2,	PRSS1
Gancao	MOL004824	(2S)-6-(2,	CCNA2
Gancao	MOL004824	(2S)-6-(2,	PCP4
Gancao	MOL004827	Semilicois	NOS2
Gancao	MOL004827	Semilicois	ESR1
Gancao	MOL004827	Semilicois	AR
Gancao	MOL004827	Semilicois	SCN5A
Gancao	MOL004827	Semilicois	PPARG
Gancao	MOL004827	Semilicois	PTGS2
Gancao	MOL004827	Semilicois	F7
Gancao	MOL004827	Semilicois	ACHE
Gancao	MOL004827	Semilicois	GSK3B

Gancao	MOL004827	Semilicois	HSP90AB1
Gancao	MOL004827	Semilicois	CDK2
Gancao	MOL004827	Semilicois	CHEK1
Gancao	MOL004827	Semilicois	PRSS1
Gancao	MOL004827	Semilicois	PCP4
Gancao	MOL004828	Glepidotir	NOS2
Gancao	MOL004828	Glepidotir	PTGS1
Gancao	MOL004828	Glepidotir	ESR1
Gancao	MOL004828	Glepidotir	AR
Gancao	MOL004828	Glepidotir	SCN5A
Gancao	MOL004828	Glepidotir	PPARG
Gancao	MOL004828	Glepidotir	PTGS2
Gancao	MOL004828	Glepidotir	F7
Gancao	MOL004828	Glepidotir	KDR
Gancao	MOL004828	Glepidotir	RXRA
Gancao	MOL004828	Glepidotir	DPP4
Gancao	MOL004828	Glepidotir	MAPK14
Gancao	MOL004828	Glepidotir	GSK3B
Gancao	MOL004828	Glepidotir	HSP90AB1
Gancao	MOL004828	Glepidotir	CDK2
Gancao	MOL004828	Glepidotir	CHEK1
Gancao	MOL004828	Glepidotir	IGHG1
Gancao	MOL004828	Glepidotir	PRSS1
Gancao	MOL004828	Glepidotir	CCNA2
Gancao	MOL004828	Glepidotir	PCP4
Gancao	MOL004829	Glepidotir	PTGS1
Gancao	MOL004829	Glepidotir	ESR1
Gancao	MOL004829	Glepidotir	SCN5A
Gancao	MOL004829	Glepidotir	PTGS2
Gancao	MOL004829	Glepidotir	F7
Gancao	MOL004829	Glepidotir	RXRA
Gancao	MOL004829	Glepidotir	ADRA1B
Gancao	MOL004829	Glepidotir	HSP90AB1
Gancao	MOL004829	Glepidotir	IGHG1
Gancao	MOL004829	Glepidotir	NCOA1
Gancao	MOL004829	Glepidotir	PCP4
Gancao	MOL004833	Phaseolini	NOS2
Gancao	MOL004833	Phaseolini	CHRM1
Gancao	MOL004833	Phaseolini	ESR1
Gancao	MOL004833	Phaseolini	AR
Gancao	MOL004833	Phaseolini	SCN5A
Gancao	MOL004833	Phaseolini	PPARG
Gancao	MOL004833	Phaseolini	PTGS2
Gancao	MOL004833	Phaseolini	RXRA
Gancao	MOL004833	Phaseolini	ACHE
Gancao	MOL004833	Phaseolini	ADRA1B
Gancao	MOL004833	Phaseolini	ADRB2
Gancao	MOL004833	Phaseolini	ESR2
Gancao	MOL004833	Phaseolini	MAPK14
Gancao	MOL004833	Phaseolini	GSK3B
Gancao	MOL004833	Phaseolini	CDK2

Gancao	MOL004833	Phaseolini	CHEK1
Gancao	MOL004833	Phaseolini	PRSS1
Gancao	MOL004833	Phaseolini	CCNA2
Gancao	MOL004833	Phaseolini	NCOA1
Gancao	MOL004833	Phaseolini	PCP4
Gancao	MOL004835	Glypallich	NOS2
Gancao	MOL004835	Glypallich	PTGS1
Gancao	MOL004835	Glypallich	CHRM1
Gancao	MOL004835	Glypallich	ESR1
Gancao	MOL004835	Glypallich	AR
Gancao	MOL004835	Glypallich	SCN5A
Gancao	MOL004835	Glypallich	PPARG
Gancao	MOL004835	Glypallich	PTGS2
Gancao	MOL004835	Glypallich	CA2
Gancao	MOL004835	Glypallich	ADRA1B
Gancao	MOL004835	Glypallich	SLC6A3
Gancao	MOL004835	Glypallich	ADRB2
Gancao	MOL004835	Glypallich	SLC6A4
Gancao	MOL004835	Glypallich	ESR2
Gancao	MOL004835	Glypallich	MAPK14
Gancao	MOL004835	Glypallich	GSK3B
Gancao	MOL004835	Glypallich	HSP90AB1
Gancao	MOL004835	Glypallich	CDK2
Gancao	MOL004835	Glypallich	LTA4H
Gancao	MOL004835	Glypallich	MAOB
Gancao	MOL004835	Glypallich	CHEK1
Gancao	MOL004835	Glypallich	CCNA2
Gancao	MOL004835	Glypallich	NCOA1
Gancao	MOL004835	Glypallich	PKIA
Gancao	MOL004835	Glypallich	PCP4
Gancao	MOL004838	8-(6-hydro	NOS2
Gancao	MOL004838	8-(6-hydro	ESR1
Gancao	MOL004838	8-(6-hydro	PTGS2
Gancao	MOL004838	8-(6-hydro	RXRA
Gancao	MOL004838	8-(6-hydro	HSP90AB1
Gancao	MOL004841	Licochalco	NOS2
Gancao	MOL004841	Licochalco	PTGS1
Gancao	MOL004841	Licochalco	ESR1
Gancao	MOL004841	Licochalco	AR
Gancao	MOL004841	Licochalco	PPARG
Gancao	MOL004841	Licochalco	PTGS2
Gancao	MOL004841	Licochalco	CA2
Gancao	MOL004841	Licochalco	ADRB2
Gancao	MOL004841	Licochalco	ESR2
Gancao	MOL004841	Licochalco	MAPK14
Gancao	MOL004841	Licochalco	GSK3B
Gancao	MOL004841	Licochalco	HSP90AB1
Gancao	MOL004841	Licochalco	CDK2
Gancao	MOL004841	Licochalco	CHEK1
Gancao	MOL004841	Licochalco	CCNA2
Gancao	MOL004841	Licochalco	PCP4

Gancao	MOL004848	licochalc	NOS2
Gancao	MOL004848	licochalc	ESR1
Gancao	MOL004848	licochalc	AR
Gancao	MOL004848	licochalc	PPARG
Gancao	MOL004848	licochalc	PTGS2
Gancao	MOL004848	licochalc	KDR
Gancao	MOL004848	licochalc	ESR2
Gancao	MOL004848	licochalc	MAPK14
Gancao	MOL004848	licochalc	GSK3B
Gancao	MOL004848	licochalc	HSP90AB1
Gancao	MOL004848	licochalc	CDK2
Gancao	MOL004848	licochalc	IGHG1
Gancao	MOL004848	licochalc	CCNA2
Gancao	MOL004848	licochalc	NCOA2
Gancao	MOL004848	licochalc	PCP4
Gancao	MOL004849	3-(2,4-di	NOS2
Gancao	MOL004849	3-(2,4-di	KCNH2
Gancao	MOL004849	3-(2,4-di	ESR1
Gancao	MOL004849	3-(2,4-di	AR
Gancao	MOL004849	3-(2,4-di	PPARG
Gancao	MOL004849	3-(2,4-di	PTGS2
Gancao	MOL004849	3-(2,4-di	F7
Gancao	MOL004849	3-(2,4-di	KDR
Gancao	MOL004849	3-(2,4-di	ESR2
Gancao	MOL004849	3-(2,4-di	DPP4
Gancao	MOL004849	3-(2,4-di	MAPK14
Gancao	MOL004849	3-(2,4-di	GSK3B
Gancao	MOL004849	3-(2,4-di	HSP90AB1
Gancao	MOL004849	3-(2,4-di	CDK2
Gancao	MOL004849	3-(2,4-di	CHEK1
Gancao	MOL004849	3-(2,4-di	PRSS1
Gancao	MOL004849	3-(2,4-di	NCOA2
Gancao	MOL004849	3-(2,4-di	NCOA1
Gancao	MOL004849	3-(2,4-di	PCP4
Gancao	MOL004855	Licoricon	NOS2
Gancao	MOL004855	Licoricon	KCNH2
Gancao	MOL004855	Licoricon	ESR1
Gancao	MOL004855	Licoricon	AR
Gancao	MOL004855	Licoricon	PPARG
Gancao	MOL004855	Licoricon	PTGS2
Gancao	MOL004855	Licoricon	KDR
Gancao	MOL004855	Licoricon	CHEK1
Gancao	MOL004855	Licoricon	PRSS1
Gancao	MOL004855	Licoricon	NCOA2
Gancao	MOL004855	Licoricon	PCP4
Gancao	MOL004856	Gancaonin	NOS2
Gancao	MOL004856	Gancaonin	ESR1
Gancao	MOL004856	Gancaonin	AR
Gancao	MOL004856	Gancaonin	SCN5A
Gancao	MOL004856	Gancaonin	PPARG
Gancao	MOL004856	Gancaonin	PTGS2

Gancao	MOL004856	Gancaonin	ACHE
Gancao	MOL004856	Gancaonin	ESR2
Gancao	MOL004856	Gancaonin	DPP4
Gancao	MOL004856	Gancaonin	GSK3B
Gancao	MOL004856	Gancaonin	HSP90AB1
Gancao	MOL004856	Gancaonin	CHEK1
Gancao	MOL004856	Gancaonin	PRSS1
Gancao	MOL004856	Gancaonin	CCNA2
Gancao	MOL004856	Gancaonin	NCOA2
Gancao	MOL004856	Gancaonin	PCP4
Gancao	MOL004857	Gancaonin	NOS2
Gancao	MOL004857	Gancaonin	ESR1
Gancao	MOL004857	Gancaonin	AR
Gancao	MOL004857	Gancaonin	PPARG
Gancao	MOL004857	Gancaonin	PTGS2
Gancao	MOL004857	Gancaonin	F7
Gancao	MOL004857	Gancaonin	KDR
Gancao	MOL004857	Gancaonin	ADRA1B
Gancao	MOL004857	Gancaonin	ADRB2
Gancao	MOL004857	Gancaonin	ESR2
Gancao	MOL004857	Gancaonin	DPP4
Gancao	MOL004857	Gancaonin	GSK3B
Gancao	MOL004857	Gancaonin	HSP90AB1
Gancao	MOL004857	Gancaonin	CHEK1
Gancao	MOL004857	Gancaonin	PRSS1
Gancao	MOL004857	Gancaonin	CCNA2
Gancao	MOL004857	Gancaonin	NCOA2
Gancao	MOL004857	Gancaonin	PCP4
Gancao	MOL004863	3-(3,4-dih	NOS2
Gancao	MOL004863	3-(3,4-dih	ESR1
Gancao	MOL004863	3-(3,4-dih	AR
Gancao	MOL004863	3-(3,4-dih	PPARG
Gancao	MOL004863	3-(3,4-dih	PTGS2
Gancao	MOL004863	3-(3,4-dih	MAPK14
Gancao	MOL004863	3-(3,4-dih	GSK3B
Gancao	MOL004863	3-(3,4-dih	HSP90AB1
Gancao	MOL004863	3-(3,4-dih	CDK2
Gancao	MOL004863	3-(3,4-dih	CHEK1
Gancao	MOL004863	3-(3,4-dih	PRSS1
Gancao	MOL004863	3-(3,4-dih	CCNA2
Gancao	MOL004863	3-(3,4-dih	NCOA2
Gancao	MOL004863	3-(3,4-dih	PCP4
Gancao	MOL004864	5,7-dihydr	NOS2
Gancao	MOL004864	5,7-dihydr	KCNH2
Gancao	MOL004864	5,7-dihydr	ESR1
Gancao	MOL004864	5,7-dihydr	AR
Gancao	MOL004864	5,7-dihydr	PPARG
Gancao	MOL004864	5,7-dihydr	PTGS2
Gancao	MOL004864	5,7-dihydr	ESR2
Gancao	MOL004864	5,7-dihydr	DPP4
Gancao	MOL004864	5,7-dihydr	MAPK14

Gancao	MOL004864	5,7-dihydr	GSK3B
Gancao	MOL004864	5,7-dihydr	HSP90AB1
Gancao	MOL004864	5,7-dihydr	CDK2
Gancao	MOL004864	5,7-dihydr	CHEK1
Gancao	MOL004864	5,7-dihydr	PRSS1
Gancao	MOL004864	5,7-dihydr	CCNA2
Gancao	MOL004864	5,7-dihydr	NCOA2
Gancao	MOL004864	5,7-dihydr	PCP4
Gancao	MOL004866	2-(3,4-dih	AR
Gancao	MOL004866	2-(3,4-dih	SCN5A
Gancao	MOL004866	2-(3,4-dih	PPARG
Gancao	MOL004866	2-(3,4-dih	PTGS2
Gancao	MOL004866	2-(3,4-dih	F7
Gancao	MOL004866	2-(3,4-dih	ADRB2
Gancao	MOL004866	2-(3,4-dih	DPP4
Gancao	MOL004866	2-(3,4-dih	HSP90AB1
Gancao	MOL004866	2-(3,4-dih	CDK2
Gancao	MOL004866	2-(3,4-dih	CHEK1
Gancao	MOL004866	2-(3,4-dih	PRSS1
Gancao	MOL004866	2-(3,4-dih	CCNA2
Gancao	MOL004866	2-(3,4-dih	PCP4
Gancao	MOL004879	Glycyrin	NOS2
Gancao	MOL004879	Glycyrin	KCNH2
Gancao	MOL004879	Glycyrin	ESR1
Gancao	MOL004879	Glycyrin	AR
Gancao	MOL004879	Glycyrin	PPARG
Gancao	MOL004879	Glycyrin	PTGS2
Gancao	MOL004879	Glycyrin	KDR
Gancao	MOL004879	Glycyrin	ESR2
Gancao	MOL004879	Glycyrin	DPP4
Gancao	MOL004879	Glycyrin	CHEK1
Gancao	MOL004879	Glycyrin	PRSS1
Gancao	MOL004879	Glycyrin	NCOA2
Gancao	MOL004879	Glycyrin	PCP4
Gancao	MOL004882	Licocoumar	ESR1
Gancao	MOL004882	Licocoumar	AR
Gancao	MOL004882	Licocoumar	ESR2
Gancao	MOL004882	Licocoumar	GSK3B
Gancao	MOL004882	Licocoumar	HSP90AB1
Gancao	MOL004882	Licocoumar	CDK2
Gancao	MOL004882	Licocoumar	CCNA2
Gancao	MOL004883	Licoisofla	NOS2
Gancao	MOL004883	Licoisofla	ESR1
Gancao	MOL004883	Licoisofla	AR
Gancao	MOL004883	Licoisofla	PPARG
Gancao	MOL004883	Licoisofla	PTGS2
Gancao	MOL004883	Licoisofla	KDR
Gancao	MOL004883	Licoisofla	DPP4
Gancao	MOL004883	Licoisofla	MAPK14
Gancao	MOL004883	Licoisofla	HSP90AB1
Gancao	MOL004883	Licoisofla	CDK2

Gancao	MOL004883	Licoisofla	CHEK1
Gancao	MOL004883	Licoisofla	PRSS1
Gancao	MOL004883	Licoisofla	CCNA2
Gancao	MOL004883	Licoisofla	NCOA2
Gancao	MOL004883	Licoisofla	PCP4
Gancao	MOL004884	Licoisofla	NOS2
Gancao	MOL004884	Licoisofla	ESR1
Gancao	MOL004884	Licoisofla	AR
Gancao	MOL004884	Licoisofla	PPARG
Gancao	MOL004884	Licoisofla	PTGS2
Gancao	MOL004884	Licoisofla	ACHE
Gancao	MOL004884	Licoisofla	ESR2
Gancao	MOL004884	Licoisofla	GSK3B
Gancao	MOL004884	Licoisofla	CDK2
Gancao	MOL004884	Licoisofla	CHEK1
Gancao	MOL004884	Licoisofla	PRSS1
Gancao	MOL004884	Licoisofla	CCNA2
Gancao	MOL004884	Licoisofla	PCP4
Gancao	MOL004885	licoisofla	NOS2
Gancao	MOL004885	licoisofla	PTGS1
Gancao	MOL004885	licoisofla	ESR1
Gancao	MOL004885	licoisofla	AR
Gancao	MOL004885	licoisofla	SCN5A
Gancao	MOL004885	licoisofla	PPARG
Gancao	MOL004885	licoisofla	PTGS2
Gancao	MOL004885	licoisofla	F7
Gancao	MOL004885	licoisofla	ACHE
Gancao	MOL004885	licoisofla	ESR2
Gancao	MOL004885	licoisofla	GSK3B
Gancao	MOL004885	licoisofla	HSP90AB1
Gancao	MOL004885	licoisofla	CDK2
Gancao	MOL004885	licoisofla	PRSS1
Gancao	MOL004885	licoisofla	CCNA2
Gancao	MOL004885	licoisofla	NCOA1
Gancao	MOL004885	licoisofla	PCP4
Gancao	MOL004891	shinpteroc	NOS2
Gancao	MOL004891	shinpteroc	PTGS1
Gancao	MOL004891	shinpteroc	CHRM3
Gancao	MOL004891	shinpteroc	KCNH2
Gancao	MOL004891	shinpteroc	CHRM1
Gancao	MOL004891	shinpteroc	ESR1
Gancao	MOL004891	shinpteroc	AR
Gancao	MOL004891	shinpteroc	SCN5A
Gancao	MOL004891	shinpteroc	PPARG
Gancao	MOL004891	shinpteroc	PTGS2
Gancao	MOL004891	shinpteroc	HTR3A
Gancao	MOL004891	shinpteroc	RXRA
Gancao	MOL004891	shinpteroc	OPRD1
Gancao	MOL004891	shinpteroc	ADRA1B
Gancao	MOL004891	shinpteroc	ADRB2
Gancao	MOL004891	shinpteroc	ADRA1D

Gancao	MOL004891	shinpteroc	OPRM1
Gancao	MOL004891	shinpteroc	ESR2
Gancao	MOL004891	shinpteroc	MAPK14
Gancao	MOL004891	shinpteroc	GSK3B
Gancao	MOL004891	shinpteroc	CDK2
Gancao	MOL004891	shinpteroc	RXRB
Gancao	MOL004891	shinpteroc	PRSS1
Gancao	MOL004891	shinpteroc	CCNA2
Gancao	MOL004891	shinpteroc	NCOA1
Gancao	MOL004891	shinpteroc	PCP4
Gancao	MOL004898	(E)-3-[3,4	ESR1
Gancao	MOL004898	(E)-3-[3,4	AR
Gancao	MOL004898	(E)-3-[3,4	PPARG
Gancao	MOL004898	(E)-3-[3,4	PTGS2
Gancao	MOL004898	(E)-3-[3,4	MAPK14
Gancao	MOL004898	(E)-3-[3,4	GSK3B
Gancao	MOL004898	(E)-3-[3,4	HSP90AB1
Gancao	MOL004898	(E)-3-[3,4	CDK2
Gancao	MOL004898	(E)-3-[3,4	CCNA2
Gancao	MOL004898	(E)-3-[3,4	NCOA2
Gancao	MOL004898	(E)-3-[3,4	PCP4
Gancao	MOL004903	liquiritin	F7
Gancao	MOL004903	liquiritin	PCP4
Gancao	MOL004903	liquiritin	PTGS2
Gancao	MOL004903	liquiritin	KDR
Gancao	MOL004903	liquiritin	SOD1
Gancao	MOL004904	licopyranc	NOS2
Gancao	MOL004904	licopyranc	ESR1
Gancao	MOL004904	licopyranc	AR
Gancao	MOL004904	licopyranc	PPARG
Gancao	MOL004904	licopyranc	PTGS2
Gancao	MOL004904	licopyranc	F7
Gancao	MOL004904	licopyranc	KDR
Gancao	MOL004904	licopyranc	ACHE
Gancao	MOL004904	licopyranc	CDK2
Gancao	MOL004904	licopyranc	PRSS1
Gancao	MOL004904	licopyranc	CCNA2
Gancao	MOL004904	licopyranc	PCP4
Gancao	MOL004907	Glyzaglabr	NOS2
Gancao	MOL004907	Glyzaglabr	PTGS1
Gancao	MOL004907	Glyzaglabr	ESR1
Gancao	MOL004907	Glyzaglabr	AR
Gancao	MOL004907	Glyzaglabr	PPARG
Gancao	MOL004907	Glyzaglabr	PTGS2
Gancao	MOL004907	Glyzaglabr	ESR2
Gancao	MOL004907	Glyzaglabr	DPP4
Gancao	MOL004907	Glyzaglabr	MAPK14
Gancao	MOL004907	Glyzaglabr	GSK3B
Gancao	MOL004907	Glyzaglabr	HSP90AB1
Gancao	MOL004907	Glyzaglabr	CDK2
Gancao	MOL004907	Glyzaglabr	CHEK1

Gancao	MOL004907	Glyzaglabr	PRSS1
Gancao	MOL004907	Glyzaglabr	CCNA2
Gancao	MOL004908	Glabridin	NOS2
Gancao	MOL004908	Glabridin	CHRM1
Gancao	MOL004908	Glabridin	ESR1
Gancao	MOL004908	Glabridin	AR
Gancao	MOL004908	Glabridin	SCN5A
Gancao	MOL004908	Glabridin	PPARG
Gancao	MOL004908	Glabridin	PTGS2
Gancao	MOL004908	Glabridin	RXRA
Gancao	MOL004908	Glabridin	ACHE
Gancao	MOL004908	Glabridin	ADRA1B
Gancao	MOL004908	Glabridin	ADRB2
Gancao	MOL004908	Glabridin	ESR2
Gancao	MOL004908	Glabridin	MAPK14
Gancao	MOL004908	Glabridin	GSK3B
Gancao	MOL004908	Glabridin	CDK2
Gancao	MOL004908	Glabridin	CHEK1
Gancao	MOL004908	Glabridin	RXRB
Gancao	MOL004908	Glabridin	IGHG1
Gancao	MOL004908	Glabridin	PRSS1
Gancao	MOL004908	Glabridin	CCNA2
Gancao	MOL004908	Glabridin	NCOA2
Gancao	MOL004908	Glabridin	NCOA1
Gancao	MOL004908	Glabridin	PCP4
Gancao	MOL004910	Glabranin	NOS2
Gancao	MOL004910	Glabranin	PTGS1
Gancao	MOL004910	Glabranin	ESR1
Gancao	MOL004910	Glabranin	SCN5A
Gancao	MOL004910	Glabranin	PTGS2
Gancao	MOL004910	Glabranin	HSP90AB1
Gancao	MOL004910	Glabranin	PCP4
Gancao	MOL004911	Glabrene	NOS2
Gancao	MOL004911	Glabrene	PTGS1
Gancao	MOL004911	Glabrene	ESR1
Gancao	MOL004911	Glabrene	AR
Gancao	MOL004911	Glabrene	SCN5A
Gancao	MOL004911	Glabrene	PPARG
Gancao	MOL004911	Glabrene	PTGS2
Gancao	MOL004911	Glabrene	RXRA
Gancao	MOL004911	Glabrene	ADRB2
Gancao	MOL004911	Glabrene	ESR2
Gancao	MOL004911	Glabrene	MAPK14
Gancao	MOL004911	Glabrene	GSK3B
Gancao	MOL004911	Glabrene	HSP90AB1
Gancao	MOL004911	Glabrene	CDK2
Gancao	MOL004911	Glabrene	PRSS1
Gancao	MOL004911	Glabrene	NCOA2
Gancao	MOL004911	Glabrene	PCP4
Gancao	MOL004912	Glabrone	NOS2
Gancao	MOL004912	Glabrone	PTGS1

Gancao	MOL004912	Glabrone	ESR1
Gancao	MOL004912	Glabrone	AR
Gancao	MOL004912	Glabrone	SCN5A
Gancao	MOL004912	Glabrone	PPARG
Gancao	MOL004912	Glabrone	PTGS2
Gancao	MOL004912	Glabrone	RXRA
Gancao	MOL004912	Glabrone	ACHE
Gancao	MOL004912	Glabrone	ESR2
Gancao	MOL004912	Glabrone	DPP4
Gancao	MOL004912	Glabrone	MAPK14
Gancao	MOL004912	Glabrone	GSK3B
Gancao	MOL004912	Glabrone	CDK2
Gancao	MOL004912	Glabrone	CHEK1
Gancao	MOL004912	Glabrone	PRSS1
Gancao	MOL004912	Glabrone	CCNA2
Gancao	MOL004912	Glabrone	PCP4
Gancao	MOL004913	1,3-dihydr	ESR1
Gancao	MOL004913	1,3-dihydr	PPARG
Gancao	MOL004913	1,3-dihydr	ESR2
Gancao	MOL004913	1,3-dihydr	MAPK14
Gancao	MOL004913	1,3-dihydr	GSK3B
Gancao	MOL004913	1,3-dihydr	HSP90AB1
Gancao	MOL004913	1,3-dihydr	CDK2
Gancao	MOL004913	1,3-dihydr	CHEK1
Gancao	MOL004913	1,3-dihydr	CCNA2
Gancao	MOL004914	1,3-dihydr	ESR1
Gancao	MOL004914	1,3-dihydr	AR
Gancao	MOL004914	1,3-dihydr	PPARG
Gancao	MOL004914	1,3-dihydr	MAPK14
Gancao	MOL004914	1,3-dihydr	GSK3B
Gancao	MOL004914	1,3-dihydr	HSP90AB1
Gancao	MOL004914	1,3-dihydr	CDK2
Gancao	MOL004914	1,3-dihydr	CHEK1
Gancao	MOL004915	Eurycarpir	NOS2
Gancao	MOL004915	Eurycarpir	ESR1
Gancao	MOL004915	Eurycarpir	AR
Gancao	MOL004915	Eurycarpir	SCN5A
Gancao	MOL004915	Eurycarpir	PPARG
Gancao	MOL004915	Eurycarpir	PTGS2
Gancao	MOL004915	Eurycarpir	ESR2
Gancao	MOL004915	Eurycarpir	DPP4
Gancao	MOL004915	Eurycarpir	MAPK14
Gancao	MOL004915	Eurycarpir	GSK3B
Gancao	MOL004915	Eurycarpir	HSP90AB1
Gancao	MOL004915	Eurycarpir	CDK2
Gancao	MOL004915	Eurycarpir	CHEK1
Gancao	MOL004915	Eurycarpir	PRSS1
Gancao	MOL004915	Eurycarpir	CCNA2
Gancao	MOL004915	Eurycarpir	PCP4
Gancao	MOL004924	(-)-Medic	PTGS2
Gancao	MOL004924	(-)-Medic	ACHE

Gancao	MOL004935	Sigmoidin-	ESR1
Gancao	MOL004935	Sigmoidin-	PTGS2
Gancao	MOL004935	Sigmoidin-	KDR
Gancao	MOL004935	Sigmoidin-	HSP90AB1
Gancao	MOL004935	Sigmoidin-	PCP4
Gancao	MOL004941	(2R)-7-hyd	PTGS1
Gancao	MOL004941	(2R)-7-hyd	ESR1
Gancao	MOL004941	(2R)-7-hyd	PTGS2
Gancao	MOL004941	(2R)-7-hyd	RXRA
Gancao	MOL004941	(2R)-7-hyd	ADRB2
Gancao	MOL004941	(2R)-7-hyd	HSP90AB1
Gancao	MOL004941	(2R)-7-hyd	DPEP1
Gancao	MOL004941	(2R)-7-hyd	MAOB
Gancao	MOL004941	(2R)-7-hyd	PKIA
Gancao	MOL004941	(2R)-7-hyd	PCP4
Gancao	MOL004941	(2R)-7-hyd	GABRA1
Gancao	MOL004941	(2R)-7-hyd	SLC6A4
Gancao	MOL004945	(2S)-7-hyd	NOS2
Gancao	MOL004945	(2S)-7-hyd	PTGS1
Gancao	MOL004945	(2S)-7-hyd	ESR1
Gancao	MOL004945	(2S)-7-hyd	SCN5A
Gancao	MOL004945	(2S)-7-hyd	PTGS2
Gancao	MOL004945	(2S)-7-hyd	ADRA1B
Gancao	MOL004945	(2S)-7-hyd	ADRB2
Gancao	MOL004945	(2S)-7-hyd	ESR2
Gancao	MOL004945	(2S)-7-hyd	HSP90AB1
Gancao	MOL004945	(2S)-7-hyd	PCP4
Gancao	MOL004948	Isoglycyro	NOS2
Gancao	MOL004948	Isoglycyro	ESR1
Gancao	MOL004948	Isoglycyro	AR
Gancao	MOL004948	Isoglycyro	PTGS2
Gancao	MOL004948	Isoglycyro	DPP4
Gancao	MOL004948	Isoglycyro	GSK3B
Gancao	MOL004949	Isolicofla	NOS2
Gancao	MOL004949	Isolicofla	ESR1
Gancao	MOL004949	Isolicofla	AR
Gancao	MOL004949	Isolicofla	PPARG
Gancao	MOL004949	Isolicofla	PTGS2
Gancao	MOL004949	Isolicofla	GSK3B
Gancao	MOL004949	Isolicofla	HSP90AB1
Gancao	MOL004949	Isolicofla	CDK2
Gancao	MOL004949	Isolicofla	PRSS1
Gancao	MOL004949	Isolicofla	CCNA2
Gancao	MOL004949	Isolicofla	NCOA2
Gancao	MOL004949	Isolicofla	PCP4
Gancao	MOL004957	HMO	NOS2
Gancao	MOL004957	HMO	PTGS1
Gancao	MOL004957	HMO	CHRM1
Gancao	MOL004957	HMO	ESR1
Gancao	MOL004957	HMO	AR
Gancao	MOL004957	HMO	SCN5A

Gancao	MOL004957	HMO	PPARG
Gancao	MOL004957	HMO	PTGS2
Gancao	MOL004957	HMO	RXRA
Gancao	MOL004957	HMO	SLC6A3
Gancao	MOL004957	HMO	ADRB2
Gancao	MOL004957	HMO	SLC6A4
Gancao	MOL004957	HMO	ESR2
Gancao	MOL004957	HMO	DPP4
Gancao	MOL004957	HMO	MAPK14
Gancao	MOL004957	HMO	GSK3B
Gancao	MOL004957	HMO	CDK2
Gancao	MOL004957	HMO	MAOB
Gancao	MOL004957	HMO	CHEK1
Gancao	MOL004957	HMO	IGHG1
Gancao	MOL004957	HMO	PRSS1
Gancao	MOL004957	HMO	CCNA2
Gancao	MOL004957	HMO	PKIA
Gancao	MOL004957	HMO	PCP4
Gancao	MOL004959	1-Methoxyp	NOS2
Gancao	MOL004959	1-Methoxyp	PTGS1
Gancao	MOL004959	1-Methoxyp	KCNH2
Gancao	MOL004959	1-Methoxyp	ESR1
Gancao	MOL004959	1-Methoxyp	AR
Gancao	MOL004959	1-Methoxyp	SCN5A
Gancao	MOL004959	1-Methoxyp	PPARG
Gancao	MOL004959	1-Methoxyp	PTGS2
Gancao	MOL004959	1-Methoxyp	KDR
Gancao	MOL004959	1-Methoxyp	RXRA
Gancao	MOL004959	1-Methoxyp	ADRA1B
Gancao	MOL004959	1-Methoxyp	ADRB2
Gancao	MOL004959	1-Methoxyp	ADRA1D
Gancao	MOL004959	1-Methoxyp	ESR2
Gancao	MOL004959	1-Methoxyp	MAPK14
Gancao	MOL004959	1-Methoxyp	GSK3B
Gancao	MOL004959	1-Methoxyp	HSP90AB1
Gancao	MOL004959	1-Methoxyp	CDK2
Gancao	MOL004959	1-Methoxyp	PRSS1
Gancao	MOL004959	1-Methoxyp	CCNA2
Gancao	MOL004959	1-Methoxyp	NCOA2
Gancao	MOL004959	1-Methoxyp	NCOA1
Gancao	MOL004959	1-Methoxyp	PCP4
Gancao	MOL004961	Quercetin	NOS2
Gancao	MOL004961	Quercetin	PTGS1
Gancao	MOL004961	Quercetin	ESR1
Gancao	MOL004961	Quercetin	AR
Gancao	MOL004961	Quercetin	SCN5A
Gancao	MOL004961	Quercetin	PPARG
Gancao	MOL004961	Quercetin	PTGS2
Gancao	MOL004961	Quercetin	ESR2
Gancao	MOL004961	Quercetin	DPP4
Gancao	MOL004961	Quercetin	MAPK14

Gancao	MOL004961	Quercetin	GSK3B
Gancao	MOL004961	Quercetin	HSP90AB1
Gancao	MOL004961	Quercetin	CDK2
Gancao	MOL004961	Quercetin	PRSS1
Gancao	MOL004961	Quercetin	NCOA2
Gancao	MOL004961	Quercetin	PCP4
Gancao	MOL004966	3'-Hydroxy	NOS2
Gancao	MOL004966	3'-Hydroxy	PTGS1
Gancao	MOL004966	3'-Hydroxy	KCNH2
Gancao	MOL004966	3'-Hydroxy	ESR1
Gancao	MOL004966	3'-Hydroxy	AR
Gancao	MOL004966	3'-Hydroxy	SCN5A
Gancao	MOL004966	3'-Hydroxy	PPARG
Gancao	MOL004966	3'-Hydroxy	PTGS2
Gancao	MOL004966	3'-Hydroxy	F7
Gancao	MOL004966	3'-Hydroxy	KDR
Gancao	MOL004966	3'-Hydroxy	ADRA1B
Gancao	MOL004966	3'-Hydroxy	ADRB2
Gancao	MOL004966	3'-Hydroxy	ESR2
Gancao	MOL004966	3'-Hydroxy	MAPK14
Gancao	MOL004966	3'-Hydroxy	GSK3B
Gancao	MOL004966	3'-Hydroxy	HSP90AB1
Gancao	MOL004966	3'-Hydroxy	CDK2
Gancao	MOL004966	3'-Hydroxy	CHEK1
Gancao	MOL004966	3'-Hydroxy	PRSS1
Gancao	MOL004966	3'-Hydroxy	CCNA2
Gancao	MOL004966	3'-Hydroxy	NCOA2
Gancao	MOL004966	3'-Hydroxy	NCOA1
Gancao	MOL004966	3'-Hydroxy	PCP4
Gancao	MOL000497	licochalco	NOS2
Gancao	MOL000497	licochalco	PTGS1
Gancao	MOL000497	licochalco	CHRM1
Gancao	MOL000497	licochalco	ESR1
Gancao	MOL000497	licochalco	AR
Gancao	MOL000497	licochalco	SCN5A
Gancao	MOL000497	licochalco	PPARG
Gancao	MOL000497	licochalco	PTGS2
Gancao	MOL000497	licochalco	CA2
Gancao	MOL000497	licochalco	ADRA1B
Gancao	MOL000497	licochalco	SLC6A3
Gancao	MOL000497	licochalco	ESR2
Gancao	MOL000497	licochalco	MAPK14
Gancao	MOL000497	licochalco	GSK3B
Gancao	MOL000497	licochalco	HSP90AB1
Gancao	MOL000497	licochalco	CDK2
Gancao	MOL000497	licochalco	CHEK1
Gancao	MOL000497	licochalco	CCNA2
Gancao	MOL000497	licochalco	PCP4
Gancao	MOL000497	licochalco	ADRB2
Gancao	MOL000497	licochalco	NCOA2
Gancao	MOL000497	licochalco	RELA

Gancao	MOL000497	licochalco	STAT3
Gancao	MOL000497	licochalco	CCND1
Gancao	MOL000497	licochalco	BCL2
Gancao	MOL000497	licochalco	EIF6
Gancao	MOL000497	licochalco	MAPK1
Gancao	MOL000497	licochalco	RB1
Gancao	MOL000497	licochalco	CDK4
Gancao	MOL000497	licochalco	FOSL2
Gancao	MOL004974	3'-Methoxy	NOS2
Gancao	MOL004974	3'-Methoxy	PTGS1
Gancao	MOL004974	3'-Methoxy	KCNH2
Gancao	MOL004974	3'-Methoxy	ESR1
Gancao	MOL004974	3'-Methoxy	AR
Gancao	MOL004974	3'-Methoxy	SCN5A
Gancao	MOL004974	3'-Methoxy	PPARG
Gancao	MOL004974	3'-Methoxy	PTGS2
Gancao	MOL004974	3'-Methoxy	F7
Gancao	MOL004974	3'-Methoxy	RXRA
Gancao	MOL004974	3'-Methoxy	ACHE
Gancao	MOL004974	3'-Methoxy	ADRA1B
Gancao	MOL004974	3'-Methoxy	ADRB2
Gancao	MOL004974	3'-Methoxy	ESR2
Gancao	MOL004974	3'-Methoxy	MAPK14
Gancao	MOL004974	3'-Methoxy	GSK3B
Gancao	MOL004974	3'-Methoxy	HSP90AB1
Gancao	MOL004974	3'-Methoxy	CDK2
Gancao	MOL004974	3'-Methoxy	CHEK1
Gancao	MOL004974	3'-Methoxy	PRSS1
Gancao	MOL004974	3'-Methoxy	CCNA2
Gancao	MOL004974	3'-Methoxy	NCOA2
Gancao	MOL004974	3'-Methoxy	NCOA1
Gancao	MOL004974	3'-Methoxy	PCP4
Gancao	MOL004978	2-[(3R)-8,	NOS2
Gancao	MOL004978	2-[(3R)-8,	PTGS1
Gancao	MOL004978	2-[(3R)-8,	CHRM3
Gancao	MOL004978	2-[(3R)-8,	KCNH2
Gancao	MOL004978	2-[(3R)-8,	CHRM1
Gancao	MOL004978	2-[(3R)-8,	ESR1
Gancao	MOL004978	2-[(3R)-8,	AR
Gancao	MOL004978	2-[(3R)-8,	SCN5A
Gancao	MOL004978	2-[(3R)-8,	PPARG
Gancao	MOL004978	2-[(3R)-8,	PTGS2
Gancao	MOL004978	2-[(3R)-8,	RXRA
Gancao	MOL004978	2-[(3R)-8,	ACHE
Gancao	MOL004978	2-[(3R)-8,	ADRA1B
Gancao	MOL004978	2-[(3R)-8,	SLC6A3
Gancao	MOL004978	2-[(3R)-8,	ADRB2
Gancao	MOL004978	2-[(3R)-8,	ESR2
Gancao	MOL004978	2-[(3R)-8,	MAPK14
Gancao	MOL004978	2-[(3R)-8,	GSK3B
Gancao	MOL004978	2-[(3R)-8,	CDK2

Gancao	MOL004978	2-[(3R)-8,	CHEK1
Gancao	MOL004978	2-[(3R)-8,	RXRB
Gancao	MOL004978	2-[(3R)-8,	PRSS1
Gancao	MOL004978	2-[(3R)-8,	CCNA2
Gancao	MOL004978	2-[(3R)-8,	NCOA2
Gancao	MOL004978	2-[(3R)-8,	NCOA1
Gancao	MOL004978	2-[(3R)-8,	PCP4
Gancao	MOL004980	Inflacouma	ESR1
Gancao	MOL004980	Inflacouma	AR
Gancao	MOL004980	Inflacouma	PPARG
Gancao	MOL004980	Inflacouma	PTGS2
Gancao	MOL004980	Inflacouma	ADRB2
Gancao	MOL004980	Inflacouma	DPP4
Gancao	MOL004980	Inflacouma	HSP90AB1
Gancao	MOL004980	Inflacouma	PRSS1
Gancao	MOL004980	Inflacouma	NCOA2
Gancao	MOL004980	Inflacouma	PCP4
Gancao	MOL004980	Inflacouma	PTGS1
Gancao	MOL004980	Inflacouma	SCN5A
Gancao	MOL004985	icos-5-enc	NCOA2
Gancao	MOL004988	Kanzonol F	ESR1
Gancao	MOL004988	Kanzonol F	AR
Gancao	MOL004988	Kanzonol F	PTGS2
Gancao	MOL004988	Kanzonol F	ESR2
Gancao	MOL004988	Kanzonol F	NCOA2
Gancao	MOL004988	Kanzonol F	PCP4
Gancao	MOL004989	6-prenylat	NOS2
Gancao	MOL004989	6-prenylat	ESR1
Gancao	MOL004989	6-prenylat	SCN5A
Gancao	MOL004989	6-prenylat	PTGS2
Gancao	MOL004989	6-prenylat	F7
Gancao	MOL004989	6-prenylat	HSP90AB1
Gancao	MOL004989	6-prenylat	PCP4
Gancao	MOL004990	7,2',4'-tr	NOS2
Gancao	MOL004990	7,2',4'-tr	PTGS1
Gancao	MOL004990	7,2',4'-tr	ESR1
Gancao	MOL004990	7,2',4'-tr	AR
Gancao	MOL004990	7,2',4'-tr	PPARG
Gancao	MOL004990	7,2',4'-tr	PTGS2
Gancao	MOL004990	7,2',4'-tr	ESR2
Gancao	MOL004990	7,2',4'-tr	DPP4
Gancao	MOL004990	7,2',4'-tr	MAPK14
Gancao	MOL004990	7,2',4'-tr	GSK3B
Gancao	MOL004990	7,2',4'-tr	HSP90AB1
Gancao	MOL004990	7,2',4'-tr	CDK2
Gancao	MOL004990	7,2',4'-tr	CHEK1
Gancao	MOL004991	7-Acetoxy-	NOS2
Gancao	MOL004991	7-Acetoxy-	PTGS1
Gancao	MOL004991	7-Acetoxy-	ESR1
Gancao	MOL004991	7-Acetoxy-	AR
Gancao	MOL004991	7-Acetoxy-	SCN5A

Gancao	MOL004991	7-Acetoxy-	PPARG
Gancao	MOL004991	7-Acetoxy-	PTGS2
Gancao	MOL004991	7-Acetoxy-	RXRA
Gancao	MOL004991	7-Acetoxy-	ACHE
Gancao	MOL004991	7-Acetoxy-	ADRA1B
Gancao	MOL004991	7-Acetoxy-	ADRB2
Gancao	MOL004991	7-Acetoxy-	ADRA1D
Gancao	MOL004991	7-Acetoxy-	GABRA1
Gancao	MOL004991	7-Acetoxy-	DPP4
Gancao	MOL004991	7-Acetoxy-	MAPK14
Gancao	MOL004991	7-Acetoxy-	GSK3B
Gancao	MOL004991	7-Acetoxy-	HSP90AB1
Gancao	MOL004991	7-Acetoxy-	CDK2
Gancao	MOL004991	7-Acetoxy-	CHEK1
Gancao	MOL004991	7-Acetoxy-	PRSS1
Gancao	MOL004991	7-Acetoxy-	NCOA2
Gancao	MOL004991	7-Acetoxy-	PCP4
Gancao	MOL004993	8-prenylat	ESR1
Gancao	MOL004993	8-prenylat	SCN5A
Gancao	MOL004993	8-prenylat	PTGS2
Gancao	MOL004993	8-prenylat	F7
Gancao	MOL004993	8-prenylat	HSP90AB1
Gancao	MOL004993	8-prenylat	NCOA1
Gancao	MOL004993	8-prenylat	PCP4
Gancao	MOL004996	gadelaidic	NCOA2
Gancao	MOL000500	Vestitol	NOS2
Gancao	MOL000500	Vestitol	PTGS1
Gancao	MOL000500	Vestitol	CHRM1
Gancao	MOL000500	Vestitol	ESR1
Gancao	MOL000500	Vestitol	AR
Gancao	MOL000500	Vestitol	SCN5A
Gancao	MOL000500	Vestitol	PPARG
Gancao	MOL000500	Vestitol	PTGS2
Gancao	MOL000500	Vestitol	CHRM4
Gancao	MOL000500	Vestitol	RXRA
Gancao	MOL000500	Vestitol	ADRA1A
Gancao	MOL000500	Vestitol	ADRA1B
Gancao	MOL000500	Vestitol	SLC6A3
Gancao	MOL000500	Vestitol	ADRB2
Gancao	MOL000500	Vestitol	SLC6A4
Gancao	MOL000500	Vestitol	ESR2
Gancao	MOL000500	Vestitol	DPP4
Gancao	MOL000500	Vestitol	MAPK14
Gancao	MOL000500	Vestitol	GSK3B
Gancao	MOL000500	Vestitol	HSP90AB1
Gancao	MOL000500	Vestitol	CDK2
Gancao	MOL000500	Vestitol	CHEK1
Gancao	MOL000500	Vestitol	PRSS1
Gancao	MOL000500	Vestitol	CCNA2
Gancao	MOL000500	Vestitol	PKIA
Gancao	MOL000500	Vestitol	PCP4

Gancao	MOL005000	Gancaonin	NOS2
Gancao	MOL005000	Gancaonin	ESR1
Gancao	MOL005000	Gancaonin	AR
Gancao	MOL005000	Gancaonin	PPARG
Gancao	MOL005000	Gancaonin	PTGS2
Gancao	MOL005000	Gancaonin	ESR2
Gancao	MOL005000	Gancaonin	DPP4
Gancao	MOL005000	Gancaonin	MAPK14
Gancao	MOL005000	Gancaonin	GSK3B
Gancao	MOL005000	Gancaonin	HSP90AB1
Gancao	MOL005000	Gancaonin	CHEK1
Gancao	MOL005000	Gancaonin	PRSS1
Gancao	MOL005000	Gancaonin	CCNA2
Gancao	MOL005000	Gancaonin	NCOA2
Gancao	MOL005000	Gancaonin	PCP4
Gancao	MOL005001	Gancaonin	ESR1
Gancao	MOL005001	Gancaonin	AR
Gancao	MOL005001	Gancaonin	PTGS2
Gancao	MOL005001	Gancaonin	KDR
Gancao	MOL005001	Gancaonin	HSP90AB1
Gancao	MOL005001	Gancaonin	PRSS1
Gancao	MOL005001	Gancaonin	CCNA2
Gancao	MOL005001	Gancaonin	NCOA2
Gancao	MOL005001	Gancaonin	PCP4
Gancao	MOL005003	Licoagroca	NOS2
Gancao	MOL005003	Licoagroca	PTGS1
Gancao	MOL005003	Licoagroca	CHRM3
Gancao	MOL005003	Licoagroca	KCNH2
Gancao	MOL005003	Licoagroca	CHRM1
Gancao	MOL005003	Licoagroca	ESR1
Gancao	MOL005003	Licoagroca	AR
Gancao	MOL005003	Licoagroca	SCN5A
Gancao	MOL005003	Licoagroca	PPARG
Gancao	MOL005003	Licoagroca	CHRM5
Gancao	MOL005003	Licoagroca	PTGS2
Gancao	MOL005003	Licoagroca	RXRA
Gancao	MOL005003	Licoagroca	ACHE
Gancao	MOL005003	Licoagroca	ADRA1B
Gancao	MOL005003	Licoagroca	ADRB2
Gancao	MOL005003	Licoagroca	ESR2
Gancao	MOL005003	Licoagroca	MAPK14
Gancao	MOL005003	Licoagroca	GSK3B
Gancao	MOL005003	Licoagroca	HSP90AB1
Gancao	MOL005003	Licoagroca	CDK2
Gancao	MOL005003	Licoagroca	RXRB
Gancao	MOL005003	Licoagroca	PRSS1
Gancao	MOL005003	Licoagroca	CCNA2
Gancao	MOL005003	Licoagroca	NCOA2
Gancao	MOL005003	Licoagroca	PCP4
Gancao	MOL005007	Glyasperir	NOS2
Gancao	MOL005007	Glyasperir	PTGS1

Gancao	MOL005007	Glyasperir	KCNH2
Gancao	MOL005007	Glyasperir	ESR1
Gancao	MOL005007	Glyasperir	AR
Gancao	MOL005007	Glyasperir	SCN5A
Gancao	MOL005007	Glyasperir	PPARG
Gancao	MOL005007	Glyasperir	PTGS2
Gancao	MOL005007	Glyasperir	F7
Gancao	MOL005007	Glyasperir	KDR
Gancao	MOL005007	Glyasperir	ACHE
Gancao	MOL005007	Glyasperir	ESR2
Gancao	MOL005007	Glyasperir	PPARD
Gancao	MOL005007	Glyasperir	GSK3B
Gancao	MOL005007	Glyasperir	HSP90AB1
Gancao	MOL005007	Glyasperir	CDK2
Gancao	MOL005007	Glyasperir	PRSS1
Gancao	MOL005007	Glyasperir	CCNA2
Gancao	MOL005007	Glyasperir	NCOA2
Gancao	MOL005007	Glyasperir	NCOA1
Gancao	MOL005007	Glyasperir	PCP4
Gancao	MOL005008	Glycyrrhiz	NOS2
Gancao	MOL005008	Glycyrrhiz	ESR1
Gancao	MOL005008	Glycyrrhiz	AR
Gancao	MOL005008	Glycyrrhiz	PTGS2
Gancao	MOL005008	Glycyrrhiz	F7
Gancao	MOL005008	Glycyrrhiz	ACHE
Gancao	MOL005008	Glycyrrhiz	ESR2
Gancao	MOL005008	Glycyrrhiz	DPP4
Gancao	MOL005008	Glycyrrhiz	GSK3B
Gancao	MOL005008	Glycyrrhiz	HSP90AB1
Gancao	MOL005008	Glycyrrhiz	CDK2
Gancao	MOL005008	Glycyrrhiz	PRSS1
Gancao	MOL005008	Glycyrrhiz	CCNA2
Gancao	MOL005008	Glycyrrhiz	PCP4
Gancao	MOL005012	Licoagrois	NOS2
Gancao	MOL005012	Licoagrois	ESR1
Gancao	MOL005012	Licoagrois	AR
Gancao	MOL005012	Licoagrois	SCN5A
Gancao	MOL005012	Licoagrois	PPARG
Gancao	MOL005012	Licoagrois	PTGS2
Gancao	MOL005012	Licoagrois	ESR2
Gancao	MOL005012	Licoagrois	DPP4
Gancao	MOL005012	Licoagrois	MAPK14
Gancao	MOL005012	Licoagrois	GSK3B
Gancao	MOL005012	Licoagrois	CDK2
Gancao	MOL005012	Licoagrois	CHEK1
Gancao	MOL005012	Licoagrois	PRSS1
Gancao	MOL005012	Licoagrois	CCNA2
Gancao	MOL005012	Licoagrois	PCP4
Gancao	MOL005016	Odoratin	NOS2
Gancao	MOL005016	Odoratin	PTGS1
Gancao	MOL005016	Odoratin	ESR1

Gancao	MOL005016	Odoratin	AR
Gancao	MOL005016	Odoratin	SCN5A
Gancao	MOL005016	Odoratin	PPARG
Gancao	MOL005016	Odoratin	PTGS2
Gancao	MOL005016	Odoratin	RXRA
Gancao	MOL005016	Odoratin	ESR2
Gancao	MOL005016	Odoratin	DPP4
Gancao	MOL005016	Odoratin	MAPK14
Gancao	MOL005016	Odoratin	GSK3B
Gancao	MOL005016	Odoratin	HSP90AB1
Gancao	MOL005016	Odoratin	CDK2
Gancao	MOL005016	Odoratin	CHEK1
Gancao	MOL005016	Odoratin	PRSS1
Gancao	MOL005016	Odoratin	CCNA2
Gancao	MOL005016	Odoratin	NCOA2
Gancao	MOL005016	Odoratin	PCP4
Gancao	MOL005017	Phaseol	ESR1
Gancao	MOL005017	Phaseol	AR
Gancao	MOL005017	Phaseol	PPARG
Gancao	MOL005017	Phaseol	PTGS2
Gancao	MOL005017	Phaseol	KDR
Gancao	MOL005017	Phaseol	MAPK14
Gancao	MOL005017	Phaseol	GSK3B
Gancao	MOL005017	Phaseol	HSP90AB1
Gancao	MOL005017	Phaseol	CDK2
Gancao	MOL005017	Phaseol	CHEK1
Gancao	MOL005017	Phaseol	CCNA2
Gancao	MOL005018	Xambioona	NOS2
Gancao	MOL005018	Xambioona	ESR1
Gancao	MOL005018	Xambioona	PTGS2
Gancao	MOL005018	Xambioona	ESR2
Gancao	MOL005018	Xambioona	NCOA2
Gancao	MOL005018	Xambioona	PCP4
Gancao	MOL005020	dehydrogl	NOS2
Gancao	MOL005020	dehydrogl	ESR1
Gancao	MOL005020	dehydrogl	AR
Gancao	MOL005020	dehydrogl	SCN5A
Gancao	MOL005020	dehydrogl	PPARG
Gancao	MOL005020	dehydrogl	PTGS2
Gancao	MOL005020	dehydrogl	ADRB2
Gancao	MOL005020	dehydrogl	ESR2
Gancao	MOL005020	dehydrogl	MAPK14
Gancao	MOL005020	dehydrogl	HSP90AB1
Gancao	MOL005020	dehydrogl	CDK2
Gancao	MOL005020	dehydrogl	CHEK1
Gancao	MOL005020	dehydrogl	PRSS1
Gancao	MOL005020	dehydrogl	CCNA2
Gancao	MOL005020	dehydrogl	NCOA2
Gancao	MOL005020	dehydrogl	PCP4
Gancao	MOL000098	quercetin	PTGS1
Gancao	MOL000098	quercetin	AR

Gancao	MOL000098	quercetin	PPARG
Gancao	MOL000098	quercetin	PTGS2
Gancao	MOL000098	quercetin	HSP90AB1
Gancao	MOL000098	quercetin	NCOA2
Gancao	MOL000098	quercetin	DPP4
Gancao	MOL000098	quercetin	AKR1B1
Gancao	MOL000098	quercetin	PRSS1
Gancao	MOL000098	quercetin	KCNH2
Gancao	MOL000098	quercetin	SCN5A
Gancao	MOL000098	quercetin	ADRB2
Gancao	MOL000098	quercetin	MMP3
Gancao	MOL000098	quercetin	F7
Gancao	MOL000098	quercetin	RXRA
Gancao	MOL000098	quercetin	ACHE
Gancao	MOL000098	quercetin	GABRA1
Gancao	MOL000098	quercetin	MAOB
Gancao	MOL000098	quercetin	RELA
Gancao	MOL000098	quercetin	EGFR
Gancao	MOL000098	quercetin	AKT1
Gancao	MOL000098	quercetin	VEGFA
Gancao	MOL000098	quercetin	CCND1
Gancao	MOL000098	quercetin	BCL2
Gancao	MOL000098	quercetin	BCL2L1
Gancao	MOL000098	quercetin	FOS
Gancao	MOL000098	quercetin	CDKN1A
Gancao	MOL000098	quercetin	EIF6
Gancao	MOL000098	quercetin	BAX
Gancao	MOL000098	quercetin	CASP9
Gancao	MOL000098	quercetin	PLAU
Gancao	MOL000098	quercetin	MMP2
Gancao	MOL000098	quercetin	MMP9
Gancao	MOL000098	quercetin	MAPK1
Gancao	MOL000098	quercetin	IL10RA
Gancao	MOL000098	quercetin	EGF
Gancao	MOL000098	quercetin	RB1
Gancao	MOL000098	quercetin	TNFAIP6
Gancao	MOL000098	quercetin	JUN
Gancao	MOL000098	quercetin	IL6R
Gancao	MOL000098	quercetin	AHSA1
Gancao	MOL000098	quercetin	CASP3
Gancao	MOL000098	quercetin	TP53
Gancao	MOL000098	quercetin	ELK1
Gancao	MOL000098	quercetin	NFKBIA
Gancao	MOL000098	quercetin	POR
Gancao	MOL000098	quercetin	ODC1
Gancao	MOL000098	quercetin	CASP8
Gancao	MOL000098	quercetin	TOP1
Gancao	MOL000098	quercetin	RAF1
Gancao	MOL000098	quercetin	SOD1
Gancao	MOL000098	quercetin	PRKCA
Gancao	MOL000098	quercetin	MMP1

Gancao	MOL000098	quercetin	HIF1A
Gancao	MOL000098	quercetin	STAT1
Gancao	MOL000098	quercetin	RUNX1T1
Gancao	MOL000098	quercetin	CDK1
Gancao	MOL000098	quercetin	HSPA5
Gancao	MOL000098	quercetin	ERBB2
Gancao	MOL000098	quercetin	PPARG
Gancao	MOL000098	quercetin	ACACA
Gancao	MOL000098	quercetin	HMOX1
Gancao	MOL000098	quercetin	CYP3A4
Gancao	MOL000098	quercetin	CYP1A2
Gancao	MOL000098	quercetin	CAV1
Gancao	MOL000098	quercetin	MYC
Gancao	MOL000098	quercetin	F3
Gancao	MOL000098	quercetin	GJA1
Gancao	MOL000098	quercetin	CYP1A1
Gancao	MOL000098	quercetin	ICAM1
Gancao	MOL000098	quercetin	IL1B
Gancao	MOL000098	quercetin	CCL2
Gancao	MOL000098	quercetin	SELE
Gancao	MOL000098	quercetin	VCAM1
Gancao	MOL000098	quercetin	PTGER3
Gancao	MOL000098	quercetin	CXCL8
Gancao	MOL000098	quercetin	PRKCB
Gancao	MOL000098	quercetin	BIRC5
Gancao	MOL000098	quercetin	DUOX2
Gancao	MOL000098	quercetin	NOS3
Gancao	MOL000098	quercetin	HSPB1
Gancao	MOL000098	quercetin	SULT1E1
Gancao	MOL000098	quercetin	IL2RA
Gancao	MOL000098	quercetin	NR1I2
Gancao	MOL000098	quercetin	CYP1B1
Gancao	MOL000098	quercetin	CCNB1
Gancao	MOL000098	quercetin	PLAT
Gancao	MOL000098	quercetin	THBD
Gancao	MOL000098	quercetin	SERPINE1
Gancao	MOL000098	quercetin	COL1A1
Gancao	MOL000098	quercetin	IFNG
Gancao	MOL000098	quercetin	ALOX5
Gancao	MOL000098	quercetin	IL1A
Gancao	MOL000098	quercetin	MPO
Gancao	MOL000098	quercetin	TOP2A
Gancao	MOL000098	quercetin	NCF1
Gancao	MOL000098	quercetin	ABCG2
Gancao	MOL000098	quercetin	HAS2
Gancao	MOL000098	quercetin	GSTP1
Gancao	MOL000098	quercetin	NFE2L2
Gancao	MOL000098	quercetin	NQO1
Gancao	MOL000098	quercetin	PARP1
Gancao	MOL000098	quercetin	AHR
Gancao	MOL000098	quercetin	PSMD3

Gancao	MOL000098	quercetin	SLC2A4
Gancao	MOL000098	quercetin	COL3A1
Gancao	MOL000098	quercetin	CXCL11
Gancao	MOL000098	quercetin	CXCL2
Gancao	MOL000098	quercetin	DCAF5
Gancao	MOL000098	quercetin	NR1I3
Gancao	MOL000098	quercetin	CHEK2
Gancao	MOL000098	quercetin	INSRR
Gancao	MOL000098	quercetin	CLDN4
Gancao	MOL000098	quercetin	PPARA
Gancao	MOL000098	quercetin	PPARD
Gancao	MOL000098	quercetin	HSF1
Gancao	MOL000098	quercetin	CXCL10
Gancao	MOL000098	quercetin	CHUK
Gancao	MOL000098	quercetin	SPP1
Gancao	MOL000098	quercetin	RUNX2
Gancao	MOL000098	quercetin	RASSF1
Gancao	MOL000098	quercetin	E2F1
Gancao	MOL000098	quercetin	E2F2
Gancao	MOL000098	quercetin	ACP3
Gancao	MOL000098	quercetin	CTSD
Gancao	MOL000098	quercetin	IGFBP3
Gancao	MOL000098	quercetin	IGF2
Gancao	MOL000098	quercetin	CD40LG
Gancao	MOL000098	quercetin	IRF1
Gancao	MOL000098	quercetin	ERBB3
Gancao	MOL000098	quercetin	PON1
Gancao	MOL000098	quercetin	DIO1
Gancao	MOL000098	quercetin	PCOLCE
Gancao	MOL000098	quercetin	NPEPPS
Gancao	MOL000098	quercetin	HK2
Gancao	MOL000098	quercetin	RASA1
Gancao	MOL000098	quercetin	GSTM1
Gancao	MOL000098	quercetin	GSTM2
Renshen	MOL002879	Diop	SCN5A
Renshen	MOL002879	Diop	ADRB2
Renshen	MOL002879	Diop	CHRM3
Renshen	MOL000449	Stigmaster	PGR
Renshen	MOL000449	Stigmaster	NR3C2
Renshen	MOL000449	Stigmaster	NCOA2
Renshen	MOL000449	Stigmaster	ADH1C
Renshen	MOL000449	Stigmaster	IGHG1
Renshen	MOL000449	Stigmaster	RXRA
Renshen	MOL000449	Stigmaster	NCOA1
Renshen	MOL000449	Stigmaster	PTGS1
Renshen	MOL000449	Stigmaster	PTGS2
Renshen	MOL000449	Stigmaster	ADRA2A
Renshen	MOL000449	Stigmaster	SLC6A2
Renshen	MOL000449	Stigmaster	SLC6A3
Renshen	MOL000449	Stigmaster	ADRB2
Renshen	MOL000449	Stigmaster	AKR1B1

Renshen	MOL000449	Stigmaster	PLAU
Renshen	MOL000449	Stigmaster	LTA4H
Renshen	MOL000449	Stigmaster	MAOB
Renshen	MOL000449	Stigmaster	MAOA
Renshen	MOL000449	Stigmaster	CTRB1
Renshen	MOL000449	Stigmaster	CHRM3
Renshen	MOL000449	Stigmaster	CHRM1
Renshen	MOL000449	Stigmaster	ADRB1
Renshen	MOL000449	Stigmaster	SCN5A
Renshen	MOL000449	Stigmaster	ADRA1A
Renshen	MOL000449	Stigmaster	CHRM2
Renshen	MOL000449	Stigmaster	ADRA1B
Renshen	MOL000449	Stigmaster	GABRA1
Renshen	MOL000358	beta-sitos	PGR
Renshen	MOL000358	beta-sitos	NCOA2
Renshen	MOL000358	beta-sitos	PTGS1
Renshen	MOL000358	beta-sitos	PTGS2
Renshen	MOL000358	beta-sitos	HSP90AB1
Renshen	MOL000358	beta-sitos	KCNH2
Renshen	MOL000358	beta-sitos	DRD1
Renshen	MOL000358	beta-sitos	CHRM3
Renshen	MOL000358	beta-sitos	CHRM1
Renshen	MOL000358	beta-sitos	SCN5A
Renshen	MOL000358	beta-sitos	CHRM4
Renshen	MOL000358	beta-sitos	ADRA1A
Renshen	MOL000358	beta-sitos	CHRM2
Renshen	MOL000358	beta-sitos	ADRA1B
Renshen	MOL000358	beta-sitos	ADRB2
Renshen	MOL000358	beta-sitos	CHRNA2
Renshen	MOL000358	beta-sitos	SLC6A4
Renshen	MOL000358	beta-sitos	OPRM1
Renshen	MOL000358	beta-sitos	GABRA1
Renshen	MOL000358	beta-sitos	BCL2
Renshen	MOL000358	beta-sitos	BAX
Renshen	MOL000358	beta-sitos	CASP9
Renshen	MOL000358	beta-sitos	JUN
Renshen	MOL000358	beta-sitos	CASP3
Renshen	MOL000358	beta-sitos	CASP8
Renshen	MOL000358	beta-sitos	PRKCA
Renshen	MOL000358	beta-sitos	PON1
Renshen	MOL000358	beta-sitos	MAP2
Renshen	MOL003648	Inermin	PTGS1
Renshen	MOL003648	Inermin	CHRM3
Renshen	MOL003648	Inermin	SCN5A
Renshen	MOL003648	Inermin	PTGS2
Renshen	MOL003648	Inermin	HTR3A
Renshen	MOL003648	Inermin	RXRA
Renshen	MOL003648	Inermin	ADRA1B
Renshen	MOL003648	Inermin	ADRB2
Renshen	MOL003648	Inermin	ADRA1D
Renshen	MOL003648	Inermin	SLC6A4

Renshen	MOL003648	Inermin	HSP90AB1
Renshen	MOL003648	Inermin	IGHG1
Renshen	MOL003648	Inermin	PRSS1
Renshen	MOL003648	Inermin	NCOA1
Renshen	MOL003648	Inermin	PCP4
Renshen	MOL000422	kaempferol	NOS2
Renshen	MOL000422	kaempferol	PTGS1
Renshen	MOL000422	kaempferol	AR
Renshen	MOL000422	kaempferol	PPARG
Renshen	MOL000422	kaempferol	PTGS2
Renshen	MOL000422	kaempferol	HSP90AB1
Renshen	MOL000422	kaempferol	NCOA2
Renshen	MOL000422	kaempferol	DPP4
Renshen	MOL000422	kaempferol	PRSS1
Renshen	MOL000422	kaempferol	PGR
Renshen	MOL000422	kaempferol	CHRM1
Renshen	MOL000422	kaempferol	ACHE
Renshen	MOL000422	kaempferol	SLC6A2
Renshen	MOL000422	kaempferol	CHRM2
Renshen	MOL000422	kaempferol	ADRA1B
Renshen	MOL000422	kaempferol	GABRA1
Renshen	MOL000422	kaempferol	F7
Renshen	MOL000422	kaempferol	PCP4
Renshen	MOL000422	kaempferol	RELA
Renshen	MOL000422	kaempferol	IKBKB
Renshen	MOL000422	kaempferol	AKT1
Renshen	MOL000422	kaempferol	BCL2
Renshen	MOL000422	kaempferol	BAX
Renshen	MOL000422	kaempferol	TNFAIP6
Renshen	MOL000422	kaempferol	JUN
Renshen	MOL000422	kaempferol	AHSA1
Renshen	MOL000422	kaempferol	CASP3
Renshen	MOL000422	kaempferol	MAPK8
Renshen	MOL000422	kaempferol	MMP1
Renshen	MOL000422	kaempferol	STAT1
Renshen	MOL000422	kaempferol	CDK1
Renshen	MOL000422	kaempferol	PPARG
Renshen	MOL000422	kaempferol	HMOX1
Renshen	MOL000422	kaempferol	CYP3A4
Renshen	MOL000422	kaempferol	CYP1A2
Renshen	MOL000422	kaempferol	CYP1A1
Renshen	MOL000422	kaempferol	ICAM1
Renshen	MOL000422	kaempferol	SELE
Renshen	MOL000422	kaempferol	VCAM1
Renshen	MOL000422	kaempferol	NR1I2
Renshen	MOL000422	kaempferol	CYP1B1
Renshen	MOL000422	kaempferol	ALOX5
Renshen	MOL000422	kaempferol	HAS2
Renshen	MOL000422	kaempferol	GSTP1
Renshen	MOL000422	kaempferol	AHR
Renshen	MOL000422	kaempferol	PSMD3

Renshen	MOL000422	kaempferol	SLC2A4
Renshen	MOL000422	kaempferol	NR1I3
Renshen	MOL000422	kaempferol	INSRR
Renshen	MOL000422	kaempferol	DIO1
Renshen	MOL000422	kaempferol	PPP3CA
Renshen	MOL000422	kaempferol	GSTM1
Renshen	MOL000422	kaempferol	GSTM2
Renshen	MOL000422	kaempferol	AKR1C3
Renshen	MOL000422	kaempferol	SLPI
Renshen	MOL005308	Aposiopola	CHRM3
Renshen	MOL005308	Aposiopola	CHRM1
Renshen	MOL005308	Aposiopola	SLC6A2
Renshen	MOL005308	Aposiopola	SLC6A3
Renshen	MOL005308	Aposiopola	ADRB2
Renshen	MOL005308	Aposiopola	SLC6A4
Renshen	MOL005308	Aposiopola	GABRA1
Renshen	MOL005308	Aposiopola	DPP4
Renshen	MOL005317	Deoxyharri	AR
Renshen	MOL005317	Deoxyharri	NR3C2
Renshen	MOL005318	Dianthrami	PTGS1
Renshen	MOL005318	Dianthrami	PTGS2
Renshen	MOL005318	Dianthrami	HSP90AB1
Renshen	MOL005320	arachidona	PTGS1
Renshen	MOL005320	arachidona	PTGS2
Renshen	MOL005320	arachidona	RXRG
Renshen	MOL005320	arachidona	NCOA2
Renshen	MOL005321	Frutinone	PTGS1
Renshen	MOL005321	Frutinone	AR
Renshen	MOL005321	Frutinone	SCN5A
Renshen	MOL005321	Frutinone	PPARG
Renshen	MOL005321	Frutinone	PTGS2
Renshen	MOL005321	Frutinone	RXRA
Renshen	MOL005321	Frutinone	ADRB2
Renshen	MOL005321	Frutinone	GABRA1
Renshen	MOL005321	Frutinone	DPP4
Renshen	MOL005321	Frutinone	HSP90AB1
Renshen	MOL005321	Frutinone	ACHE
Renshen	MOL005344	ginsenosic	BAX
Renshen	MOL005344	ginsenosic	TNFAIP6
Renshen	MOL005344	ginsenosic	CASP3
Renshen	MOL005344	ginsenosic	PTGS2
Renshen	MOL005344	ginsenosic	NFKBIA
Renshen	MOL005344	ginsenosic	IL1B
Renshen	MOL005344	ginsenosic	CASP1
Renshen	MOL005344	ginsenosic	IFNG
Renshen	MOL005344	ginsenosic	ADCYAP1
Renshen	MOL005344	ginsenosic	PSMG1
Renshen	MOL005344	ginsenosic	MAP2K4
Renshen	MOL005344	ginsenosic	SLC2A4
Renshen	MOL005348	Ginsenosic	NR3C2
Renshen	MOL005348	Ginsenosic	NCOA2

Renshen	MOL005356	Girinimbir	PTGS1
Renshen	MOL005356	Girinimbir	SCN5A
Renshen	MOL005356	Girinimbir	PTGS2
Renshen	MOL005356	Girinimbir	RXRA
Renshen	MOL005356	Girinimbir	ADRB2
Renshen	MOL005356	Girinimbir	GABRA1
Renshen	MOL005356	Girinimbir	NCOA2
Renshen	MOL005376	Panaxadiol	NR3C1
Renshen	MOL005384	suchilacto	KCNH2
Renshen	MOL005384	suchilacto	SCN5A
Renshen	MOL005384	suchilacto	PTGS2
Renshen	MOL005384	suchilacto	F7
Renshen	MOL005384	suchilacto	ADRB2
Renshen	MOL005384	suchilacto	HSP90AB1
Renshen	MOL005384	suchilacto	NCOA1
Renshen	MOL005384	suchilacto	PCP4
Renshen	MOL005384	suchilacto	PTGS1
Renshen	MOL005384	suchilacto	RXRA
Renshen	MOL005384	suchilacto	ADRA1D
Renshen	MOL005399	alexandrir	PGR
Renshen	MOL000787	Fumarine	PTGS1
Renshen	MOL000787	Fumarine	CHRM3
Renshen	MOL000787	Fumarine	KCNH2
Renshen	MOL000787	Fumarine	CHRM1
Renshen	MOL000787	Fumarine	SCN5A
Renshen	MOL000787	Fumarine	CHRM5
Renshen	MOL000787	Fumarine	PTGS2
Renshen	MOL000787	Fumarine	HTR3A
Renshen	MOL000787	Fumarine	F7
Renshen	MOL000787	Fumarine	CHRM4
Renshen	MOL000787	Fumarine	OPRD1
Renshen	MOL000787	Fumarine	ADRA1B
Renshen	MOL000787	Fumarine	ADRB2
Renshen	MOL000787	Fumarine	ADRA1D
Renshen	MOL000787	Fumarine	OPRM1
Renshen	MOL000787	Fumarine	HSP90AB1
Renshen	MOL000787	Fumarine	PCP4
Renshen	MOL000787	Fumarine	SLC6A4
Renshen	MOL000787	Fumarine	CACNA1S
Renshen	MOL000787	Fumarine	SLC6A3
Renshen	MOL000787	Fumarine	DRD1
Renshen	MOL000787	Fumarine	KDR
Yiyiren	MOL001323	Sitosterol	PGR
Yiyiren	MOL001323	Sitosterol	PTGS2
Yiyiren	MOL001323	Sitosterol	GABRA1
Yiyiren	MOL001323	Sitosterol	ADH1C
Yiyiren	MOL001323	Sitosterol	NR3C2
Yiyiren	MOL001494	Mandenol	PTGS1
Yiyiren	MOL001494	Mandenol	PTGS2
Yiyiren	MOL001494	Mandenol	NCOA2
Yiyiren	MOL000359	sitosterol	PGR

Yiyiren	MOL000359	sitosterol	NCOA2
Yiyiren	MOL000359	sitosterol	NR3C2
Yiyiren	MOL000449	Stigmaster	PGR
Yiyiren	MOL000449	Stigmaster	NR3C2
Yiyiren	MOL000449	Stigmaster	NCOA2
Yiyiren	MOL000449	Stigmaster	ADH1C
Yiyiren	MOL000449	Stigmaster	IGHG1
Yiyiren	MOL000449	Stigmaster	RXRA
Yiyiren	MOL000449	Stigmaster	NCOA1
Yiyiren	MOL000449	Stigmaster	PTGS1
Yiyiren	MOL000449	Stigmaster	PTGS2
Yiyiren	MOL000449	Stigmaster	ADRA2A
Yiyiren	MOL000449	Stigmaster	SLC6A2
Yiyiren	MOL000449	Stigmaster	SLC6A3
Yiyiren	MOL000449	Stigmaster	ADRB2
Yiyiren	MOL000449	Stigmaster	AKR1B1
Yiyiren	MOL000449	Stigmaster	PLAU
Yiyiren	MOL000449	Stigmaster	LTA4H
Yiyiren	MOL000449	Stigmaster	MAOB
Yiyiren	MOL000449	Stigmaster	MAOA
Yiyiren	MOL000449	Stigmaster	CTRB1
Yiyiren	MOL000449	Stigmaster	CHRM3
Yiyiren	MOL000449	Stigmaster	CHRM1
Yiyiren	MOL000449	Stigmaster	ADRB1
Yiyiren	MOL000449	Stigmaster	SCN5A
Yiyiren	MOL000449	Stigmaster	ADRA1A
Yiyiren	MOL000449	Stigmaster	CHRM2
Yiyiren	MOL000449	Stigmaster	ADRA1B
Yiyiren	MOL000449	Stigmaster	GABRA1
Yiyiren	MOL008121	2-Monoolei	NCOA2
Yiyiren	MOL000953	CLR	PGR
Yiyiren	MOL000953	CLR	NR3C2
Yiyiren	MOL000953	CLR	NCOA2
Zhebeimu	MOL001004	pelargonic	NOS2
Zhebeimu	MOL001004	pelargonic	PTGS1
Zhebeimu	MOL001004	pelargonic	AR
Zhebeimu	MOL001004	pelargonic	PPARG
Zhebeimu	MOL001004	pelargonic	PTGS2
Zhebeimu	MOL001004	pelargonic	CA2
Zhebeimu	MOL001004	pelargonic	HSP90AB1
Zhebeimu	MOL001004	pelargonic	NCOA2
Zhebeimu	MOL001004	pelargonic	RXRA
Zhebeimu	MOL001004	pelargonic	ACHE
Zhebeimu	MOL001004	pelargonic	PGR
Zhebeimu	MOL001004	pelargonic	NR3C2
Zhebeimu	MOL001004	pelargonic	NR3C1
Zhebeimu	MOL001004	pelargonic	NCOA1
Zhebeimu	MOL000358	beta-sitos	PGR
Zhebeimu	MOL000358	beta-sitos	NCOA2
Zhebeimu	MOL000358	beta-sitos	PTGS1
Zhebeimu	MOL000358	beta-sitos	PTGS2

Zhebeimu	MOL000358	beta-sitos	HSP90AB1
Zhebeimu	MOL000358	beta-sitos	KCNH2
Zhebeimu	MOL000358	beta-sitos	DRD1
Zhebeimu	MOL000358	beta-sitos	CHRM3
Zhebeimu	MOL000358	beta-sitos	CHRM1
Zhebeimu	MOL000358	beta-sitos	SCN5A
Zhebeimu	MOL000358	beta-sitos	CHRM4
Zhebeimu	MOL000358	beta-sitos	ADRA1A
Zhebeimu	MOL000358	beta-sitos	CHRM2
Zhebeimu	MOL000358	beta-sitos	ADRA1B
Zhebeimu	MOL000358	beta-sitos	ADRB2
Zhebeimu	MOL000358	beta-sitos	CHRNA2
Zhebeimu	MOL000358	beta-sitos	SLC6A4
Zhebeimu	MOL000358	beta-sitos	OPRM1
Zhebeimu	MOL000358	beta-sitos	GABRA1
Zhebeimu	MOL000358	beta-sitos	BCL2
Zhebeimu	MOL000358	beta-sitos	BAX
Zhebeimu	MOL000358	beta-sitos	CASP9
Zhebeimu	MOL000358	beta-sitos	JUN
Zhebeimu	MOL000358	beta-sitos	CASP3
Zhebeimu	MOL000358	beta-sitos	CASP8
Zhebeimu	MOL000358	beta-sitos	PRKCA
Zhebeimu	MOL000358	beta-sitos	PON1
Zhebeimu	MOL000358	beta-sitos	MAP2
Zhebeimu	MOL004440	Peimisine	NR3C2
Zhebeimu	MOL004440	Peimisine	NR3C1
Zhebeimu	MOL004443	Zhebeiresi	PTGS1
Zhebeimu	MOL004443	Zhebeiresi	SCN5A
Zhebeimu	MOL004443	Zhebeiresi	PTGS2
Zhebeimu	MOL004443	Zhebeiresi	RXRA
Zhebeimu	MOL004443	Zhebeiresi	ADRB2
Zhebeimu	MOL004443	Zhebeiresi	GABRA1
Zhebeimu	MOL004443	Zhebeiresi	HSP90AB1
Zhebeimu	MOL004446	6-Methoxyl	ESR1
Zhebeimu	MOL004446	6-Methoxyl	PTGS2
Zhebeimu	MOL004446	6-Methoxyl	CA2
Zhebeimu	MOL004446	6-Methoxyl	HSP90AB1
Zhebeimu	MOL004446	6-Methoxyl	NCOA2
