# Supplementary material for: Network Pharmacology Approach to Investigate the Mechanism of Modified Liu Jun Zi Decoction in the Treatment of Chronic Atrophic Gastritis
Source: Evid Based Complement Alternat Med. 2022 Jun 17;2022:7536042. doi: 10.1155/2022/7536042 (PMC9232340; doi:10.1155/2022/7536042)
Supplement: Supplementary Materials — Supplementary material 1: Supplementary Table S1. Potential targets information of MLD. Supplementary material 2: Supplementary Table S2. Potential targets information of CAG. Supplementary material 3: Supplementary Table S3. Ingredients of MLD for CAG treatment. Supplementary material 4: Supplementary Table S4. Targets of MLD for CAG treatment. [file 7536042.f1.zip › 7536042.f1/Supplementary Table S2. Potential targets information of CAG.pdf]

Gene Symbol
DRD2
CYP3A4
CYP3A5
CYP3A7
ABCB1
CYP1A2
CYP2B6
CYP2C8
CYP2D6
DRD3
HRH2
SLC22A2
SLC22A8
SLC47A1
SLC22A6
PGA5
EGF
FGF2
FGA
FGB
IL1B
IL1RN
GAST
PGA3
PGA4
AIRE
CHGA
CXCL8
CDH1
TNF
IL10
KRAS
PGC
CDX2
ODC1
GHRL
S100A8
ERCC6
IL11
TP53
HLA-DRB1
PTGS2
ADH7
TFF2
CAT
RUNX3
STAT3
TFF3
SST
MUC1

MUC6
IL6
CTSW
CTNNA1
CTLA4
MAP3K6
LRBA
MEN1
GAD2
FUT2
MUC2
CBLIF
SCT
SH2D1A
ATP4A
MUC5AC
MTR
TCN2
DDC
GAD1
ALK
TH
ALB
CBL
CASR
CYP11A1
CYP17A1
TPO
PTPN22
BCL6
CYP21A2
CCR6
PTPRN
TPH1
ATP12A
TNFRSF25
TIA1
TNFRSF8
TG
CCK
CD5
TCN1
MUC4
TRAPPC10
CD52
MMD
NUDT10
MUC3A
IGH
TRB
LEPQTL1

TGFA
ERCC8
GSTP1
TGFB1
LTA
PSCA
ALDH2
OGG1
SOD1
MTHFR
PPARG
NOS2
MIR27A
SLURP1
JRK
LY6K
THEM6
lnc-JRK-1
AREG
HBEGF
PCNA
AICDA
HLA-DQB1
BCL2
NKX2-1
BGLAP
ABO
FOXJ1
MIRLET7A1
MIR429
HSPB1
FAS
MIF
TLR1
XDH
SPP1
IL1A
FUT3
HLA-DQA1
ADA
ACE
GHR
PRKAA1
ERCC4
ERCC5
DDB2
XPC
GH1
GAB1
MUC5B
NDUFS5

RNF180
SAPCD2
MUTYH
MSH2
GKN2
MCM6
CASP10
PIK3CA
APC
IRF1
KLF6
FGFR2
ATP4B
ERBB2
NTRK2
NTRK1
NTRK3
BDNF
LIPF
GRP
GIPR
ADGRL3
GRPR
GDNF
IL6R
DMD
CNTFR
LIFR
IL6ST
CNTF
NDNF
FLACC1
CDNF
MANF
NCF4
SNTG1
NTF4
SNTB1
SNTB2
ALS5
ALS3
ANGPTL8
C2CD6
NENF
SNTG2
ADGRL2
ADGRL4
CARF
ARTN
RAPH1
GFRA2

ADGRL1
SNTA1
GFRA1
TEF
ATN1
PLEKHG4
SGCA
CLCF1
NMU
MYH6
STXBP2
SLC26A7
GIP
SGCB
HADHA
KMT2E
SGCG
UTRN
SGCD
ALS2
MTPN
DAG1
CCKBR
DMPK
NTF3
ELANE
CRHR2
CCKAR
PLA2G1B
CXCR2
IFNG
OPA2
NM
OPA4
OPA5
PBCRA1
CLAM
OXTR
SCA18
NTF6G
NTF6B
NTF6A
CRHR1
LUZP6
SERF1A
DMWD
PRAM1
NCF1C
TROAP
DEFA3
OXT

LCN2
OPA3
CXCL3
IL17C
BEST4
TRO
SSPN
OAT
MAGEE1
PRG3
DRP2
PRTN3
BEST2
MMP8
TBCE
FKRP
BEST3
AOAH
CTF1
PTN
MYO1F
SGCE
SLC22A17
CKLF
BEST1
PPBP
S100A12
NCF1
PGM5
SAA1
NCF2
STXBP3
ANXA3
MIB2
PREX1
NCF1B
ITGAM
MSTN
CX3CL1
ITGA1
FCER1G
AZU1
DTNA
VAMP7
ITGB2
EDN2
OPA1
INPP5D
CCL2
NGFR
EDN3

PRKCA
CD44
CAV3
EDN1
SYK
IKZF1
RPS6KB1
ADAM17
TGFB2
CHRM5
