# Supplementary material for: Network Pharmacology Approach to Investigate the Mechanism of Modified Liu Jun Zi Decoction in the Treatment of Chronic Atrophic Gastritis
Source: Evid Based Complement Alternat Med. 2022 Jun 17;2022:7536042. doi: 10.1155/2022/7536042 (PMC9232340; doi:10.1155/2022/7536042)
Supplement: Supplementary Materials — Supplementary material 1: Supplementary Table S1. Potential targets information of MLD. Supplementary material 2: Supplementary Table S2. Potential targets information of CAG. Supplementary material 3: Supplementary Table S3. Ingredients of MLD for CAG treatment. Supplementary material 4: Supplementary Table S4. Targets of MLD for CAG treatment. [file 7536042.f1.zip › 7536042.f1/Supplementary Table S3. ingredients of MLD for CAG treatment.pdf]

MOL004810
MOL004824
MOL001484
MOL004891
MOL000787
MOL004833
MOL005000
MOL004941
MOL005008
MOL000239
MOL004914
MOL000422
MOL001323
MOL004991
MOL004924
MOL005016
MOL004910
MOL005828
MOL004820
MOL004814
MOL005100
MOL004828
MOL004806
MOL004841
MOL003656
MOL005020
MOL004829
MOL004835
MOL004908
MOL004328
MOL000392
MOL004855
MOL005320
MOL004935
MOL004808
MOL005318
MOL001792
MOL005018
MOL004904
MOL004446
MOL004961
MOL004911
MOL000358
MOL004907
MOL004966
MOL004990
MOL004885
MOL004848
MOL004866
MOL004811
MOL004898

MOL004948
MOL005017
MOL000354
MOL005001
MOL005344
MOL002311
MOL000022
MOL004949
MOL004864
MOL005007
MOL002714
MOL004883
MOL004849
MOL006967
MOL004827
MOL005321
MOL000497
MOL000072
MOL004913
MOL004805
MOL000098
MOL005356
MOL004443
MOL003896
MOL002670
MOL005003
MOL005012
MOL006957
MOL004838
MOL001494
MOL001004
MOL000049
MOL004915
MOL005815
MOL004912
MOL004903
MOL004815
MOL004959
MOL004856
MOL002565
MOL000449
MOL004879
MOL000500
MOL000296
MOL000417
MOL003648
MOL004993
MOL000519
MOL005384
MOL004989
MOL004988

MOL004863
MOL004980
MOL004978
MOL004945
MOL004957
MOL004884
MOL004857
MOL004974
