# Supplementary material for: Network Pharmacology Approach to Investigate the Mechanism of Modified Liu Jun Zi Decoction in the Treatment of Chronic Atrophic Gastritis
Source: Evid Based Complement Alternat Med. 2022 Jun 17;2022:7536042. doi: 10.1155/2022/7536042 (PMC9232340; doi:10.1155/2022/7536042)
Supplement: Supplementary Materials — Supplementary material 1: Supplementary Table S1. Potential targets information of MLD. Supplementary material 2: Supplementary Table S2. Potential targets information of CAG. Supplementary material 3: Supplementary Table S3. Ingredients of MLD for CAG treatment. Supplementary material 4: Supplementary Table S4. Targets of MLD for CAG treatment. [file 7536042.f1.zip › 7536042.f1/Supplementary Table S4. Targets of MLD for CAG treatment.pdf]

Gene Symbol
PTGS2
CHRM5
BCL2
TP53
PRKCA
PPARG
SOD1
CAT
GSTP1
NOS2
NCF1
CYP3A4
CYP1A2
STAT3
EGF
IL6R
ODC1
ERBB2
IL1B
CCL2
CXCL8
HSPB1
IFNG
IL1A
SPP1
IRF1
